# Interferon-γ-induced p27KIP1 binds to and targets MYC for proteasome-mediated degradation

**DOI:** 10.18632/oncotarget.6693

**Published:** 2015-12-20

**Authors:** Fuad Bahram, Per Hydbring, Susanna Tronnersjö, Siti Mariam Zakaria, Oliver Frings, Sara Fahlén, Helén Nilsson, Jacob Goodwin, Natalie von der Lehr, Yingtao Su, Bernhard Lüscher, Alina Castell, Lars-Gunnar Larsson

**Affiliations:** ^1^ Department of Microbiology, Tumor and Cell Biology (MTC), Karolinska Institutet, Stockholm, Sweden; ^2^ Department of Plant Biology and Forest Genetics, Swedish University of Agricultural Sciences, Uppsala, Sweden; ^3^ Department of Oncology-Pathology, Science for Life Laboratory, Karolinska Institutet, Stockholm, Sweden; ^4^ Institute of Biochemistry and Molecular Biology, Medical School, RWTH Aachen University, Aachen, Germany; ^5^ Moreinx AB, Uppsala, Sweden; ^6^ Department of Cancer Biology, Dana-Farber Cancer Institute, Boston, MA, USA; ^7^ GE Healthcare, Uppsala, Sweden; ^8^ Department of Neuroscience, Swedish Medical Nanoscience Center, Karolinska Institutet, Stockholm, Sweden; ^9^ Center for Molecular Pathology, Lund University, Lund, Sweden; ^10^ NatScience, Uppsala, Sweden; ^11^ Anxun International Co., Limited, Hong Kong, China

**Keywords:** oncogene, tumor suppressor gene, ubiquitin-proteasome system, interferon-gamma, the cancer genome atlas

## Abstract

The Myc oncoprotein is tightly regulated at multiple levels including ubiquitin-mediated protein turnover. We recently demonstrated that inhibition of Cdk2-mediated phosphorylation of Myc at Ser-62 pharmacologically or through interferon (IFN)-γ-induced expression of p27^Kip1^ (p27) repressed Myc's activity to suppress cellular senescence and differentiation. In this study we identified an additional activity of p27 to interfere with Myc independent of Ser-62 phosphorylation. p27 is required and sufficient for IFN-γ-induced turnover of Myc. p27 interacted with Myc in the nucleus involving the C-termini of the two proteins, including Myc box 4 of Myc. The C-terminus but not the Cdk2 binding fragment of p27 was sufficient for inducing Myc degradation. Protein expression data of The Cancer Genome Atlas breast invasive carcinoma set revealed significantly lower Myc protein levels in tumors with highly expressed p27 lacking phosphorylation at Thr-157 - a marker for active p27 localized in the nucleus. Further, these conditions correlated with favorable tumor stage and patient outcome. This novel regulation of Myc by IFN-γ/p27^KIP1^ potentially offers new possibilities for therapeutic intervention in tumors with deregulated Myc.

## INTRODUCTION

Oncogenes of the *Myc* family, c-*Myc*, N-*Myc* and L-*Myc*, are master regulators of cell proliferation [1–5]. The Myc genes encode basic region/helix-loop-helix/leucine zipper (bHLHZip)-type of transcription factors, which activate transcription as part of a binary complex with the bHLHZip partner protein Max. The Myc:Max complex binds to DNA sequences termed E-boxes and stimulates transcription of a large group of specific genes, which in turn influences global RNA and protein synthesis [6–9]. In addition, Myc represses many genes by interacting with the zinc finger protein Miz1 [1–5, 10]. In this way Myc coordinates multiple fundamental cellular processes, including cell cycle progression, cell growth, apoptosis, senescence, metabolism and stem cell functions. When deregulated, Myc drives oncogenic transformation and is associated with a variety of human cancers [1–5, 11].

The Myc protein is short-lived, having a half-life of around 30 min and is degraded through the ubiquitin-proteasome pathway [12]. Phosphorylation of Thr-58 within the evolutionary conserved Myc box 1 (MB1) is an important determinant of Myc stability [13, 14]. This phosphorylation is carried out by glycogen synthase kinase 3β (GSK3β) and is dependent on prior phosphorylation of Ser-62, which is mediated by Ras-MAPK signaling through Erk and by cyclin-dependent kinases (Cdks) [12, 15–17]. Whereas phosphorylation of Ser-62 alone is reported to stabilize Myc [17, 18], subsequent phosphorylation of Thr-58 promotes Myc ubiquitylation and degradation through binding of the E3 ubiquitin ligase SCF^Fbxw7^ [19, 20]. The Myc protein is stabilized in many tumors due to loss of Fbxw7, inactivation of GSK3β via deregulation of the PI3K/Akt pathway or mutation of Thr-58 [12]. In addition, Myc degradation and/or activity are regulated by several other E3 ubiquitin ligases, including SCF^Skp2^, Huwe1/HectH9, SCF^β-TrCP^ and SCF^Fbxo28^ [12, 21–24].

We recently demonstrated that Cdk2-mediated phosphorylation of Myc at Ser-62 plays an important role in regulating a subset of Myc target genes involved in suppression of cellular senescence and differentiation, thereby contributing to malignant transformation [15, 25]. Senescence induction and differentiation could be restored by pharmacological inhibition of Cdk2 or interferon-γ (IFN-γ)-induced expression of the cellular Cdk2 inhibitor p27^Kip1^, from now on referred to as p27. Here we show that p27 in addition to its inhibition of Cdk2 also overrides Myc's suppression of senescence independent of Ser-62 and induces Myc degradation independent of Cdk2 by binding Myc via its C-terminus. Further, high expression of active p27 protein significantly correlated with low Myc protein level in human breast cancer.

## RESULTS

### p27 overrides Myc's suppression of senescence independently of Myc Ser-62 status

We have previously reported that Myc cooperates with Ras in transformation of primary rat embryo fibroblasts (REFs) by suppressing Ras-induced senescence, and that this function of Myc depends on Cdk2-mediated phosphorylation of Ser-62 [15]. Further, pharmacological inhibitors of Cdk2 or enforced expression of p27 restored Ras-induced senescence. These studies predicted that expression of a Ser-62 phospho-mimicking Myc mutant would override the activity of Cdk2 inhibitors and of p27.

**Figure 1:**
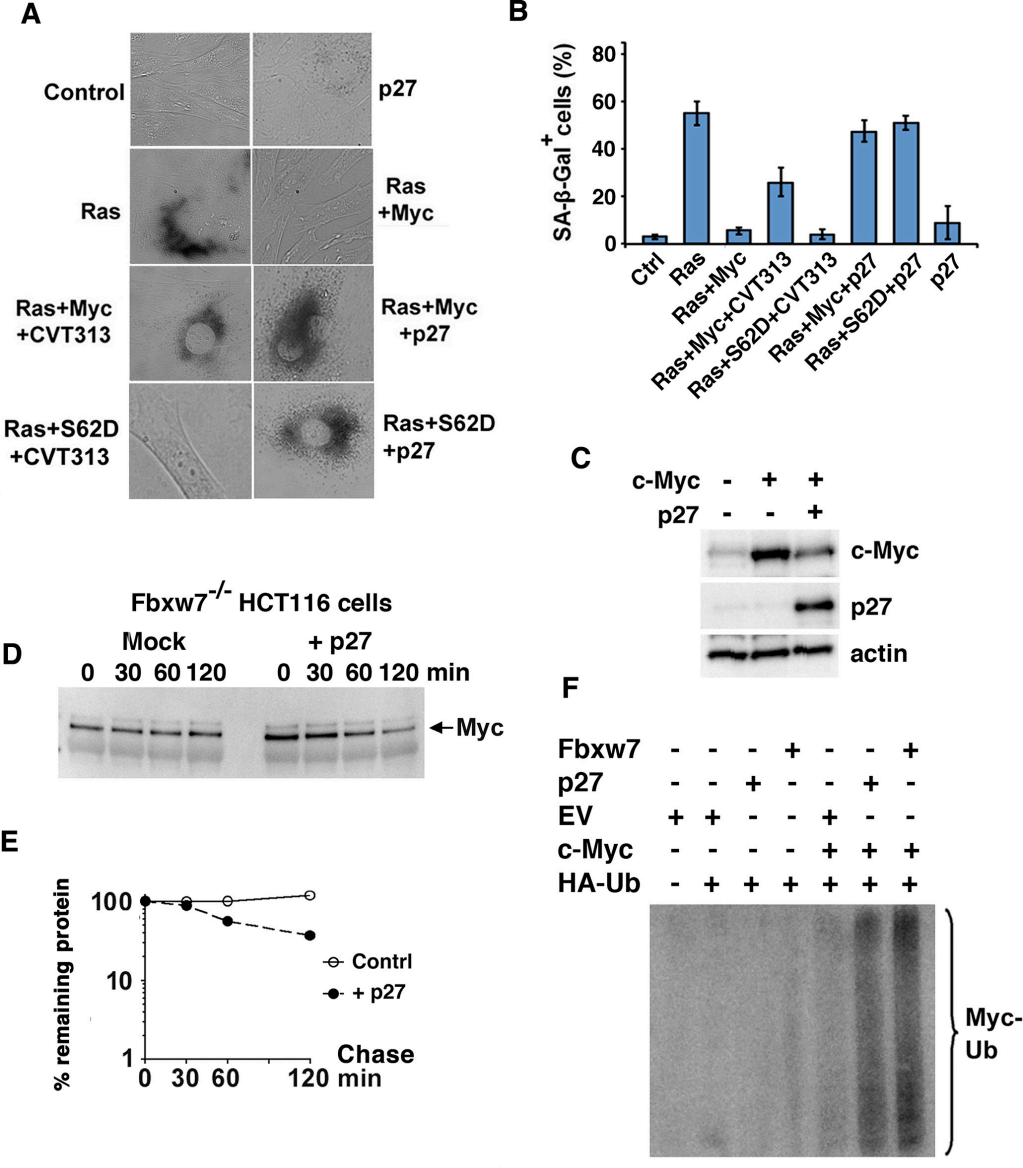
p27 overrides Myc's suppression of senescence independently of Myc Ser-62 status and induces ubiquitin-mediated degradation of the Myc protein (**A**, **B**) p27 induces senescence in Myc+Ras-transformed rat embryo fibroblasts (REFs) independent on Ser-62 status. Primary rat embryo fibroblasts (REFs) were transfected with indicated expression plasmids and treated with the CDK2 inhibitor CVT-313 or vehicle. (A), micrographs showing transfected cells cultured for 5 days before analysis of senescence by SA-β-Gal staining. (B), mean values and standard deviations of SA-β-Gal staining of at least three experiments are given based on analysis of 50 randomly chosen cells. (**C**) Ectopic expression of p27 downregulates c-Myc. HA-tagged c-Myc was cotransfected with p27 into HeLa cells. Western blot (WB) analysis of total cell lysates was carried out using HA antibodies (upper panel), p27 BD Ab (middle panel) and actin Ab (lower panel). (**D**, **E**) p27 or a control vector was transfected into HCT116 Fbxw7/Cdc4^−/−^ cells. (D) Myc turnover was determined by CHX chase by adding CHX for the indicated times before harvest and WB analysis (E) Quantification of the analysis in (D) was performed using a CCD camera. (**F**) p27 increases ubiquitylation of c-Myc. U2OS cells were transfected using the expression vectors indicated and the cells were treated with MG115. HA-Ub conjugated proteins were immunoprecipitated with HA antibodies and the captured proteins were analyzed by western blot using pan-Myc antibodies. EV = empty vector. Fbxw7 expression vector was used as reference.

To address this issue, primary REFs were cotransfected with H-Ras together with wt Myc or a phospho-mimicking Myc S62D mutant and exposed to the Cdk2 inhibitor CVT-313 or enforced expression of p27, after which senescence was measured. Ras alone induced senescence-associated β-Gal (SA-β-Gal) activity and coexpression of c-Myc suppressed senescence induction, while treatment with CVT-313 or p27 cotransfection restored senescence as expected (Figure 1A, 1B). However, when cotransfecting a phospho-mimicking S62D mutant with Ras, CVT-313 did not restore senescence, suggesting that CVT-313 induces senescence by inhibiting Ser-62 phosphorylation mediated by Cdk2. Interestingly, enforced expression of p27 still induced senescence in the presence of Myc S62D (Figure 1A, 1B). This suggests that p27 can override Myc function also independently of Cdk2-mediated Ser-62 phosphorylation. Note that expression of p27 alone in the absence of Ras and Myc did not induce senescence.

### p27 induces ubiquitin-mediated degradation of the Myc protein

To elucidate possible mechanisms by which p27 overrides Myc function, we first studied the effect of p27 overexpression on Myc protein expression. Enforced expression of p27 reduced the expression of c-Myc in HeLa cells in cotransfection experiments in a dose-dependent manner (Figure 1C and Supplementary Figure S1A). We next investigated if enforced expression of p27 affected protein turnover and ubiquitylation of Myc. In order to avoid too high background from Fbxw7, the main E3 ubiquitin ligase targeting Myc [12, 19, 20], we utilized HCT116 *Fbxw7*^−/−^ cells, in which c-Myc was stabilized (Figure 1D, 1E and Supplementary Figure S1B). However, ectopic p27 expression enhanced c-Myc turnover without affecting c-*Myc* mRNA levels. To study ubiquitylation of c-Myc, the cells were cotransfected with expression plasmids encoding c-Myc and HA-tagged ubiquitin, together with p27, Fbxw7 or empty vector. Figure 1F shows that cotransfection with p27 efficiently stimulated c-Myc ubiquitylation almost to the same extent as Fbxw7 in this assay.

### IFN-γ increases degradation and ubiquitylation of Myc through induction of p27

**Figure 2:**
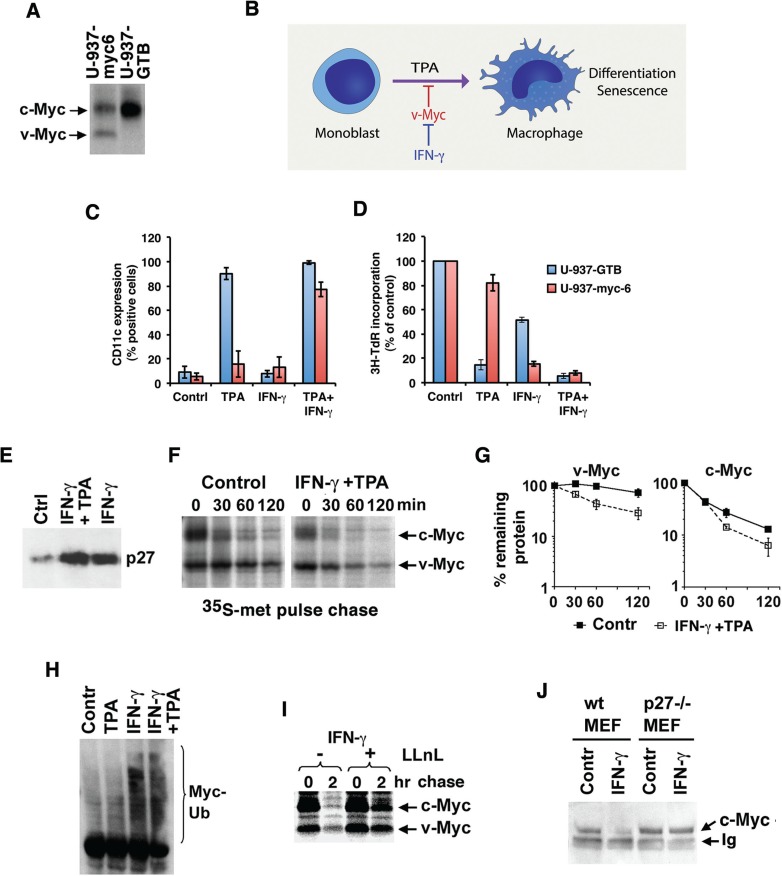
IFN-γ increases degradation and ubiquitylation of Myc through induction of p27 (**A**) Detection of v- and c-Myc in v-Myc transformed U-937-myc-6 and c-Myc in parental U-937-GTB cells after ^35^S-labeling as indicated. v-Myc migrates faster than c-Myc in SDS-PAGE gels. (**B**) A schematic picture describing the U-937 monocytic differentiation model. v-Myc blocks TPA-induced differentiation of U-937 cells and IFN-γ reverses this block. (**C** and **D**) Parental U-937-GTB and v-Myc transformed U-937-myc-6 cells were induced with TPA, IFN-γ, or TPA + IFN-γ and analyzed at day 5 after induction as indicated. (C) CD11c expression. The surface antigen expression of CD11C was measured by fluorescence-activated cell sorter analysis by using specific antibodies. (D) ^3^H-TdR incorporation. Cells were labeled with 10 mCi of ^3^H-TdR for 1 hour. (**E**) p27 expression is induced by IFN-γ. U-937-myc-6 cells were stimulated with IFN-γ or IFN-γ+TPA for 72 hrs after which p27 expression was determined by immunoblot analysis. (**F**–**G**) Combined IFN-γ and TPA treatment (24 hrs) increases degradation of v- and c-Myc in U937-myc-6 cells. v-Myc contains a point mutation at Thr-58, resulting in its stabilization. 35S-Met pulse-chase was followed by IP using a pan-Myc antiserum and SDS-PAGE. (G) Quantitation of v-Myc (left) and c-Myc (right) turnover by phosphor imager. Note the logarithmic scale of the Y-axis. (**H**) IFN-γ increases c-Myc ubiquitylation. 2fTGH cells were treated as indicated for 24 hours and cell lysates were immunoprecipitated with Myc antibodies followed by western blot using Ub antibodies. Note that endogenous proteins are analyzed and none of the components was ectopically expressed. (**I**) IFN-γ-induced Myc degradation is proteasome-dependent. Pulse chase analysis was performed in U-937-myc-6 cells treated with IFN-γ+TPA for 24 hours in the presence or absence of the proteasome inhibitor LLnL as indicated. (**J**) IFN-γ-induced degradation of c-Myc is p27-dependent. Wt and p27^−/−^ MEF cells were stimulated with murine IFN-γ for 24 hrs and the steady state level of c-Myc was determined as above.

p27 expression is induced by growth inhibitory cytokines like IFN-γ and TGF-β [15, 26–28]. We have shown previously that IFN-γ restores TPA-induced differentiation and senescence in U-937 monoblastic tumor cells overexpressing v-Myc, a viral homolog of c-Myc mutated at Thr-58 resulting in protein stabilization [15, 29] (Figure 2A, 2B for an outline of the system). As shown in Figure 2C and 2D, the induced expression of the monocytic differentiation marker CD11c and the reduced proliferation (measured as ^3^H-TdR incorporation) observed in response to TPA in parental U-937-GTB cells was strongly inhibited in U-937-myc-6 cells. However, co-stimulation with IFN-γ resulted in increased CD11c and reduced proliferation in U-937-myc-6 cells to a similar level as in TPA-treated parental cells, in accordance with previous results [15]. Treatment with IFN-γ alone did not induce differentiation neither in parental nor in U-937-myc-6 cells, but the v-Myc-expressing cells were sensitized to IFN-γ-induced growth inhibition (Figure 2C, 2D), in part due to increased senescence [15].

Both IFN-γ + TPA and IFN-γ treatments induced p27 expression in U-937-myc-6 cells (Figure 2E) in agreement with previous observations [15]. The upregulation of p27 occurred at the level of protein synthesis since the *p27* mRNA level was essentially unaffected (data not shown). We therefore addressed whether IFN-γ + TPA or IFN-γ alone might regulate v-Myc and/or endogenous c-Myc protein turnover by induction of p27. v-Myc runs slightly faster than endogenous human c-Myc in SDS-page gels [15] (compare v-Myc expressing U937-myc-6 cells and parental U-937-GTB cells in Figure 2A). Thus both v-Myc and c-Myc can be measured simultaneously.

U-937-myc-6 cells were treated with IFN-γ+TPA or left untreated for 24 hrs and then pulse/chased with ^35^S-Met (Figure 2F, 2G). In untreated cells, endogenous c-Myc had an expected short half-life of around 30 minutes. v-Myc exhibited a half-life of approximately 160 minutes as a result of the stabilizing Thr-58 mutation [13, 14]. In response to IFN-γ+TPA treatment both c-Myc and v-Myc turnover increased from approximately 30 to 20 and 150 to 50 minutes, respectively (Figure 2F, 2G). The non-linear slopes of the curves might reflect the existence of different subpopulations of Myc proteins with different stability, as reported previously [30]. TPA treatment alone did not alter c-Myc or v-Myc stability, while IFN-γ alone had similar effects on Myc turnover as had the combination IFN-γ+TPA (see below). Importantly, IFN-γ+TPA treatment did not lead to reduced v-*Myc* mRNA levels, while c-*M*yc mRNA levels decreased (Supplementary Figure S2A). TPA is known to downregulate c-*Myc* mRNA in U-937 cells [31]. Based on this we investigated whether IFN-γ would affect Myc turnover also in other cell types. Enhanced c-Myc turnover in response to IFN-γ treatment was also observed in Colo-320 colon carcinoma cells with amplified c-*Myc* (Supplementary Figure S2B, S2C) and in human 2fTGH fibrosarcoma cells (Figure 4A, 4B), showing that this phenomenon was not unique to U-937 cells.

To investigate the kinetics of IFN-γ +TPA-regulated Myc turnover we blocked de novo protein synthesis in U-937-myc-6 cells with cycloheximide (CHX) followed by chase. In response to IFN-γ + TPA, the turnover rate of v-Myc and endogenous c-Myc began to increase already within 4 hrs and was further accelerated after 8 hrs of IFN-γ + TPA treatment as demonstrated by western blot analysis (Supplementary Figure S2D, S2E). The increased v-Myc/c-Myc turnover in response to IFN-γ +TPA treatment is thus an early event that coincides with induced p27 expression and precedes effects on the cell cycle, which are not apparent until after 24 hrs of treatment [15].

As expected, the increased turnover rate of Myc in response to IFN-γ and IFN-γ + TPA was accompanied by increased Myc ubiquitylation (Figure 2H). Further, IFN-γ-induced degradation of both c- and v-Myc in U-937-myc-6 cells was inhibited by the proteasome inhibitor LLnL (Figure 2I), suggesting that the increased Myc turnover induced by this cytokine is mediated by the ubiquitin/proteasome pathway.

These observations raised the question whether p27 mediates IFN-γ-induced degradation of Myc. To clarify this, wt and p27-deficient murine embryonic fibroblasts (MEFs) were treated with IFN-γ after which Myc levels were determined by western blot analysis. While IFN-γ treatment strongly reduced Myc protein levels in wt MEFs, no effect on the steady state levels of c-Myc was seen in p27^−/−^ MEF cells, verifying that IFN-γ mediated turnover of c-Myc is indeed p27-dependent (Figure 2J). IFN-γ treatment had no effect on c-*Myc* mRNA levels in wt MEFs (Supplementary Figure S2F).

Taken together, these results suggest that IFN-γ induces Myc ubiquitin/proteasome-mediated degradation through induction of p27.

### IFN-γ-induces degradation of Myc in the nucleus in complex with Max

**Figure 3:**
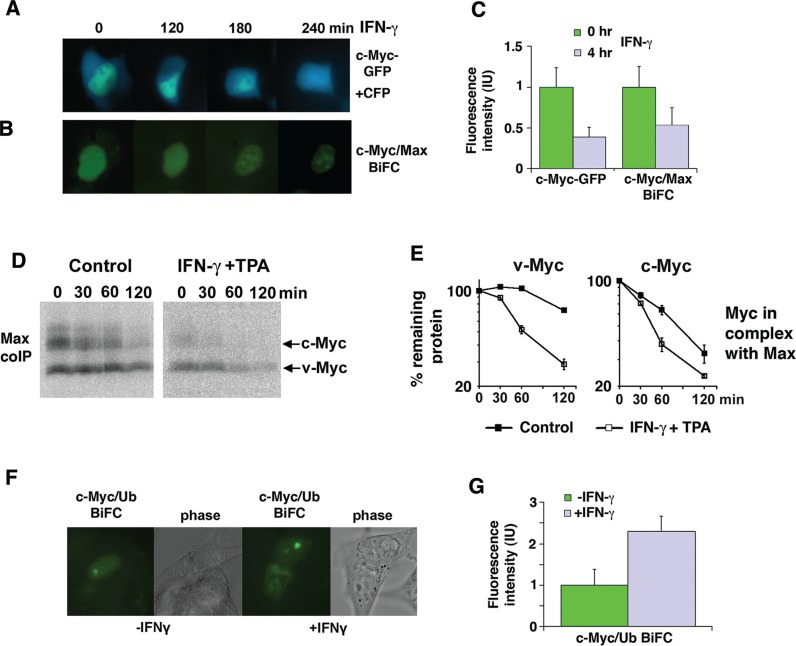
IFN-γ induces degradation of Myc in the nucleus in complex with Max (**A**–**C**) IFN-γ treatment increases c-Myc and c-Myc/Max degradation in 2fTGH cells. The micrographs show a time lapse for the indicated time points after IFN-γ treatment of 2fTGH cells expressing a c-Myc-eGFP fusion protein together with an eCFP construct (A), and a BIFC experiment (B) with c-Myc-YC and Max-YN. Quantification is shown in (C) with mean values and standard deviations of 50 cells analyzed. (**D** and **E**) Combined IFN-γ and TPA treatment increases degradation of v- and c-Myc in complex with Max in U937-myc-6 cells. ^35^S-Met pulse-chase was followed by Myc/Max coIP using α-Max C-17 antibody and SDS-PAGE. (E) Quantitation of v-Myc (left) and c-Myc (right) turnover by phosphor imager. (**F** and **G**) IFN-γ treatment increases c-Myc ubiquitylation in 2fTGH cells as determined by BiFC (c-Myc-YC and YN-Ub). (F) The micrographs show 2fTGH cells pretreated with the proteasome inhibitor MG115 followed by IFN-γ treatment for 4 hours or left untreated. Quantification is shown in G with mean values and standard deviations of 10 cells analyzed.

To study Myc turnover and intracellular localization after IFN-γ in living cells, we expressed a c-Myc-eGFP fusion protein together with an eCFP reporter protein in 2fTGH cells. IFN-γ treatment decreased the c-Myc-eGFP fluorescence signal to approximately 40% of control within 4 hrs relative to the eCFP fluorescence signal (Figure 3A, 3C). No apparent difference in the subcellular localization of a c-Myc-GFP fusion protein was observed. To determine if IFN-γ affected the steady state level of Myc:Max heterodimers, we applied Bimolecular Fluorescence Complementation (BiFC) [24, 32]. The c-Myc:Max BiFC fluorescence intensity was reduced with similar kinetics and magnitude as the c-Myc-GFP intensity after IFN-γ-stimulation (Figure 3B, 3C), suggesting that Myc was not protected from IFN-γ-mediated degradation in complex with Max. This was further confirmed by coimmunoprecipitations in U-937-myc-6 cells, showing that IFN-γ + TPA enhanced turnover of both c- and v-Myc in complex with Max (Figure 3D, 3E).

To study where ubiquitylation of Myc occurs in response to IFN-γ, a BiFC experiment was performed in living 2fTGH cells using the c-Myc-YC and YN-ubiquitin (Ub) fusion proteins to visualize conjugation of ubiquitin to c-Myc. Increased c-Myc/Ub BiFC fluorescence intensity, indicative of increased c-Myc ubiquitylation, was observed in IFN-γ stimulated compared to non-stimulated cells after proteasome inhibition (Figure 3F, 3G). The fluorescence seemed to be predominantly localized to nucleoli, suggesting that this could be a site of rapid Myc turnover, as previously suggested [33].

### IFN-γ induces degradation of Myc in a Jak/Stat-dependent but Thr-58-independent manner

**Figure 4:**
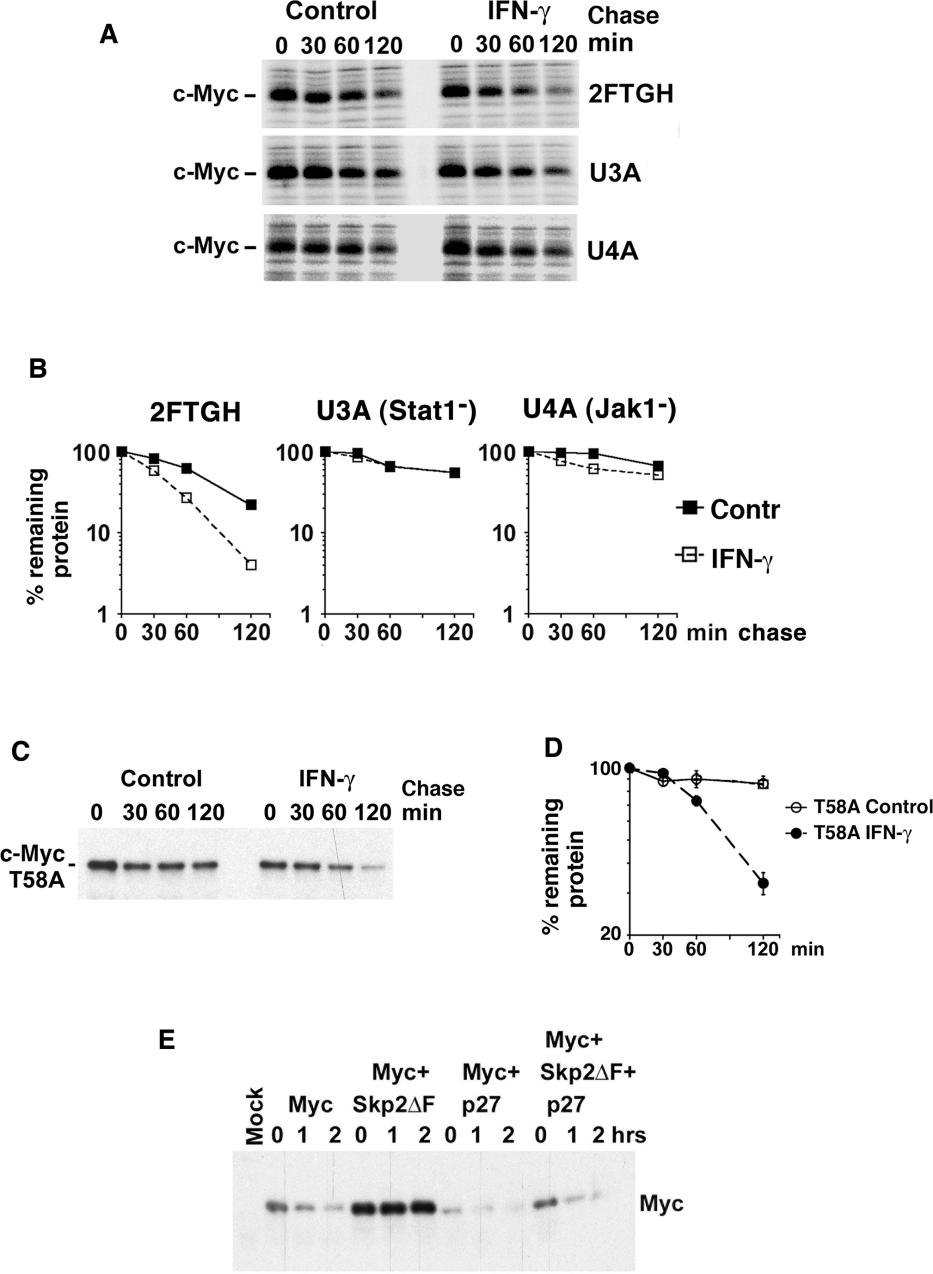
IFN-γ induces degradation of Myc in a Jak/Stat-dependent but Thr-58- and Skp2-independent manner (**A**–**B**) Parental 2fTGH fibrosarcoma cells and U3A STAT-deficient and U4A JAK1-deficient sublines were treated with IFN-γ for 24 hrs. (A) 35-S pulse chase analysis performed as in Figure 2F. (B) Quantitation of c-Myc turnover in (A) by phosphor imager. (**C**–**D**) 2fTGH cells were transfected with FLAG-tagged T58A c-Myc mutant, treated with or without IFN-γ for 24 hr, and CHX added for the indicated times. (C) c-Myc was immunoprecipitated with Flag antibodies followed by western blot analysis using pan-Myc antibodies. (D) Quantification of the analysis in (C) was performed using a CCD camera. (**E**) p27 induces Myc degradation in Skp2-independent manner. Flag-tagged c-Myc was cotransfected with p27 and/or Skp2ΔF into HeLa cells and a CHX chase experiment was performed as above.

Many of the biological effects of signaling via the IFN-γ receptor are mediated through activation of the Jak/Stat1 pathway [34]. Indeed IFN-γ treatment did not affect Myc turnover in the 2fTGH sublines U3A and U4A that lack Stat1 and Jak1, respectively, indicating that both Stat1 and Jak1 are required for IFN-γ-induced Myc degradation (Figure 4A, 4B).

As mentioned in the Introduction, the Thr-58 phosphorylation site in MB1 plays a pivotal role in the regulation of c-Myc degradation [12–14, 17, 18]. To examine whether Thr-58 is essential for IFN-γ-induced Myc degradation, the turnover of a T58A c-Myc mutant was studied by CHX chase after exogenous expression in 2fTGH cells. IFN-γ increased the rate of degradation of the c-Myc-T58A mutant, despite its low turnover rate in untreated cells (Figure 4C, 4D). This was not due to decreased c-Myc mRNA levels, which rather increased in response to IFN-γ (Supplementary Figure S3A). This is consistent with the IFN-γ effect on v-Myc (Figure 2F, 2G, S2D, S2E), which also carries a Thr-58 mutation.

Taken together these results suggest that IFN-γ-induced Myc degradation is Jak/Stat1- dependent but Myc Thr-58-independent.

### Myc stability is regulated by p27 independent of the E3 ligases SCF^Fbxw7^ and SCF^Skp2^

As shown in Figure 1, 2, 4 and Supplementary Figure S2, p27- and IFN-γ-induced degradation of c- and v-Myc is independent on Thr-58 and the E3 ubiquitin ligase subunit Fbxw7 that recognizes phosphorylated Thr-58, suggesting that IFN-γ/p27 operates via another E3 ligase. Another possible candidate was SCF^Skp2^, which promotes c-Myc degradation independent of MB1 [23, 24] and targets p27 for degradation after phosphorylation by Cdk2 [26–28]. To study if p27-induced Myc degradation required Skp2, Myc was stabilized by the dominant-negative Skp2ΔF mutant (Figure 4E). However, p27 coexpression increased Myc turnover irrespective of the presence of Skp2ΔF, suggesting that Skp2 does not participate in p27-induced Myc degradation. Notably, increased Myc turnover occurred in the presence of unchanged levels of Myc mRNA (Supplementary Figure S3B). This indicates that another, yet to be identified, E3 ligase is involved in IFN-γ/p27-induced Myc turnover.

### p27 physically interacts with c-Myc in the nucleus

**Figure 5:**
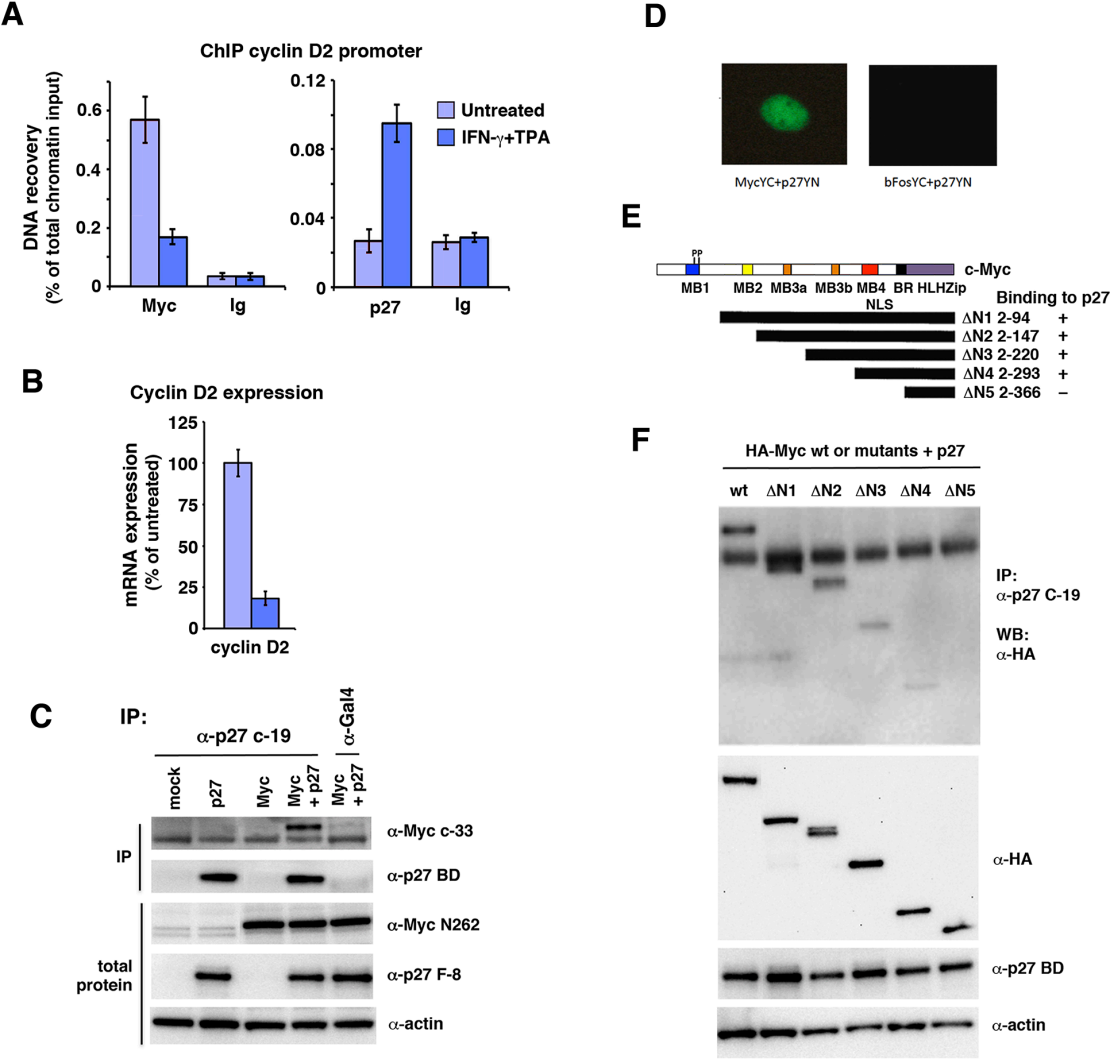
p27 associates with c-Myc bound chromatin and interacts with c-Myc through the Myc MB4-region (**A**) Q-ChIP analysis of Myc and p27 performed in untreated or IFN-γ + TPA-treated U-937-myc-6 cells using the pan-Myc (left panel) and p27 antibodies (right panel) and primers specific for an E-box-containing region of the *cyclin D2* promoter. Preimmune serum (Ig) was used as negative control. (**B**) *Cyclin D2* mRNA expression was analyzed by Q-RT-PCR in U-937-myc-6 cells treated with IFN-γ + TPA. (**C**) p27 interacts with Myc. Lysates from HCT116 Fbxw7^−/−^ cells transfected with Myc, p27 or Myc + p27 expression vectors were immunoprecipitated with p27 C-19 or Gal4 control antibodies after which immunoblot analysis was performed using pan-Myc antibodies (upper panel) and p27 BD antibodies (2nd panel from the top). Total protein in the lysates was examined with Myc N262 (3rd panel), p27 F-8 antibodies (4th panel) and actin (5th panel) antibodies. (**D**) BiFC analysis of Myc and p27 interaction. Cos7 cells were cotransfected with vectors containing c-Myc-YC and p27-YN (left panel) or bFos-YC and p27-YN as control (right panel) after which the BiFC signal was analyzed under a UV microscope. (**E**, **F**) The region around Myc MB4 is required for interactions with p27. (E), structure of c-Myc and overview of HA-tagged c-Myc deletion derivatives, and summary of interaction results. (F), CoIP analysis of interactions between p27 and indicated c-Myc deletion derivatives. wt c-Myc and mutants were cotransfected with p27 into Cos 7 cells. Upper panel, lysates were coimmunoprecipitated with p27 antibodies followed with WB using HA antibodies, 2nd–4th panel, WB analysis of total expression of wt c-Myc and mutants using HA-Myc antibodies (2nd panel), p27 (3rd panel) and actin (bottom panel).

The results above suggest that p27 targets Myc at least in part independent of inhibition of Cdk2 and Ser-62 phosphorylation. This raises the question whether p27 carries out this function by interacting with some other protein and possibly directly with Myc. We have shown previously that IFN-γ induced p27 associates with Myc target genes, correlating with their reduced expression [15]. This was confirmed by chromatin immunoprecipitation in U-937-myc-6 cells using primers covering an E-box-containing region of the Myc-regulated *cyclin D2* promoter (Figure 5A). IFN-γ + TPA stimulation led to increased binding of p27 to this region concomitant with reduced Myc binding, consistent with increased Myc turnover in response to treatment. Further, the reduced association of Myc with the *cyclin D2* promoter correlated with reduced *cyclin D2* expression as expected (Figure 5B).

The ChIP experiments along with our previous results from re-ChIP and co-immunoprecipitation experiments indicated that Myc and p27 might interact directly or indirectly [15]. To expand on these observations, p27 and c-Myc were co-expressed in HCT116 Fbxw7−/− cells followed by co-immunoprecipitation. Antibodies specific for p27 but not control antibodies co-immunoprecipitated c-Myc (Figure 5C). This supports further the suggestion that p27 and c-Myc interact.

While c-Myc is mainly localized in the nucleus, p27 is known to shuttle between the nucleus and the cytoplasm [26, 28]. To visualize the intracellular localization of the c-Myc/p27 interaction in living cells, we utilized BiFC by co-expressing Myc-YC and p27-YN fusion constructs. The c-Myc/p27 BiFC signal was localized exclusively in the nucleus, while no fluorescence was observed in control cells expressing bFosYC and p27YN (Figure 5D), indicating interaction between c-Myc and p27 in the nucleus.

### c-Myc binding to p27 requires amino acids 294-366 of Myc containing Myc box 4

To better understand the mechanism by which p27 promotes c-Myc protein turnover, we mapped the c-Myc domains interacting with p27. Binding was measured between full-length p27, and a series of N-terminal deletion mutants of Myc (Figure 5E) [35]. In these experiments, full-length c-Myc bound efficiently to p27 as described above. While constructs (ΔN1–4) lacking MB1, MB2 and MB3 still bound to p27, reduced binding was observed after removing amino acids 294–366 (ΔN5) (Figure 5E, 5F). This region contains Myc box 4 (MB4), the nuclear localization signal (NLS) and the basic DNA binding region of Myc. Hence, the region containing amino acid residues 294–366 of c-Myc is required for interaction with p27.

### The C-terminal domain of p27 interacts with c-Myc and is sufficient for its degradation

**Figure 6:**
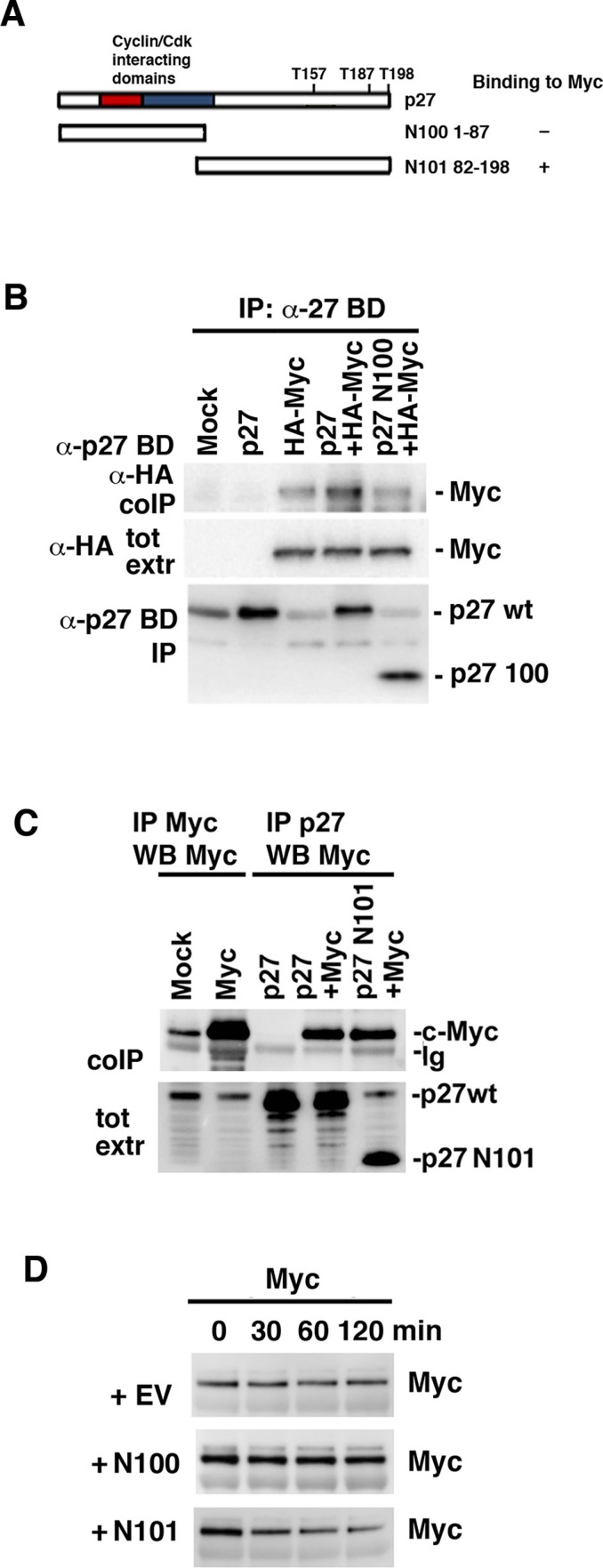
The C-terminus of p27 interacts with c-Myc and is sufficient to induce Myc degradation (**A**) Schematic representation of wt p27 structure and the two deletion mutants N100 and N101. (**B**) Myc does not bind N100. wt p27 and the N100 mutant were cotransfected with HA-Myc into Cos 7 cells. Lysates were coimmunoprecipitated with p27 BD antibodies, which recognizes the N-terminal part of p27, followed by western blot using HA antibodies (upper panel) or p27 BD antibodies (lower panel). Middle panel; western blot analysis of total expression of c-Myc using HA antibodies. (**C**) Myc binds N101. wt p27 and the N101 mutant were cotransfected with c-Myc into Cos 7 cells. Lysates were coimmunoprecipitated with Myc or p27 C-19 antibodies, which recognizes the C-terminal part of p27, followed by western blot using Myc antibodies (upper panel). Lower panel; western blot analysis of p27 total expression of using p27 C-19 antibodies. (**D**) N101 induces degradation of c-Myc. p27 N100, N101 or empty vector (EV) were transfected into HCT116 Fbxw7^−/−^ cells after which Myc turnover was determined by CHX chase.

We next investigated which part of p27 was required for interaction with c-Myc. Two p27 constructs were used in co-transfections with wt c-Myc, one covering the N-terminal 1–87 amino acids of p27 including the cyclin A/Cdk2-binding/inhibitory domain (N100) and the C-terminal part of p27 covering amino acids 82–198 (N101) (depicted in Figure 6A). The C-terminal fragment of p27 includes several regulatory phosphorylation sites. These include T187, which upon phosphorylation is recognized by Skp2 leading to degradation [26–28], and T157 and T198, which are targeted by Akt, Rsk and Pim1 and direct p27 nuclear export [26, 28] (Figure 6A). While wt p27 co-immunoprecipitated with c-Myc as expected, immunoprecipitation of the N100 fragment using antibodies directed against the N-terminal part of p27 failed to immunoprecipitate c-Myc above the level observed in cells lacking exogenous p27, which presumably represents c-Myc binding to endogenous p27 (Figure 6B). In contrast, immunoprecipitation of the N101 C-terminal fragment of p27 using antibodies directed against the C-terminal part of p27 co-immunoprecipitated c-Myc to the same extent as wt p27 (Figure 6C). This suggests that c-Myc interacts with p27 via the C-terminal region, but not via the cyclin/Cdk2-interacting region of p27.

We next investigated whether the N-terminal cyclin/Cdk2-interacting fragment or the C-terminal Myc-binding fragment contributed to Myc turnover. CHX chase analysis was performed in HCT116 Fbxw7−/− cells after transfection of the N100, N101 or empty vector (EV) constructs. N101, but not N100 or the control vector, increased c-Myc turnover (Figure 6D), indicating that p27 targets Myc for degradation by binding to c-Myc via the C-terminal part without involving binding of p27 to Cdk2. Importantly, as for wt p27, N101 did not affect c-*Myc* mRNA levels (Supplementary Figure S3C).

### High p27 expression in human breast cancer correlates with low Myc protein level and favorable outcome

**Figure 7:**
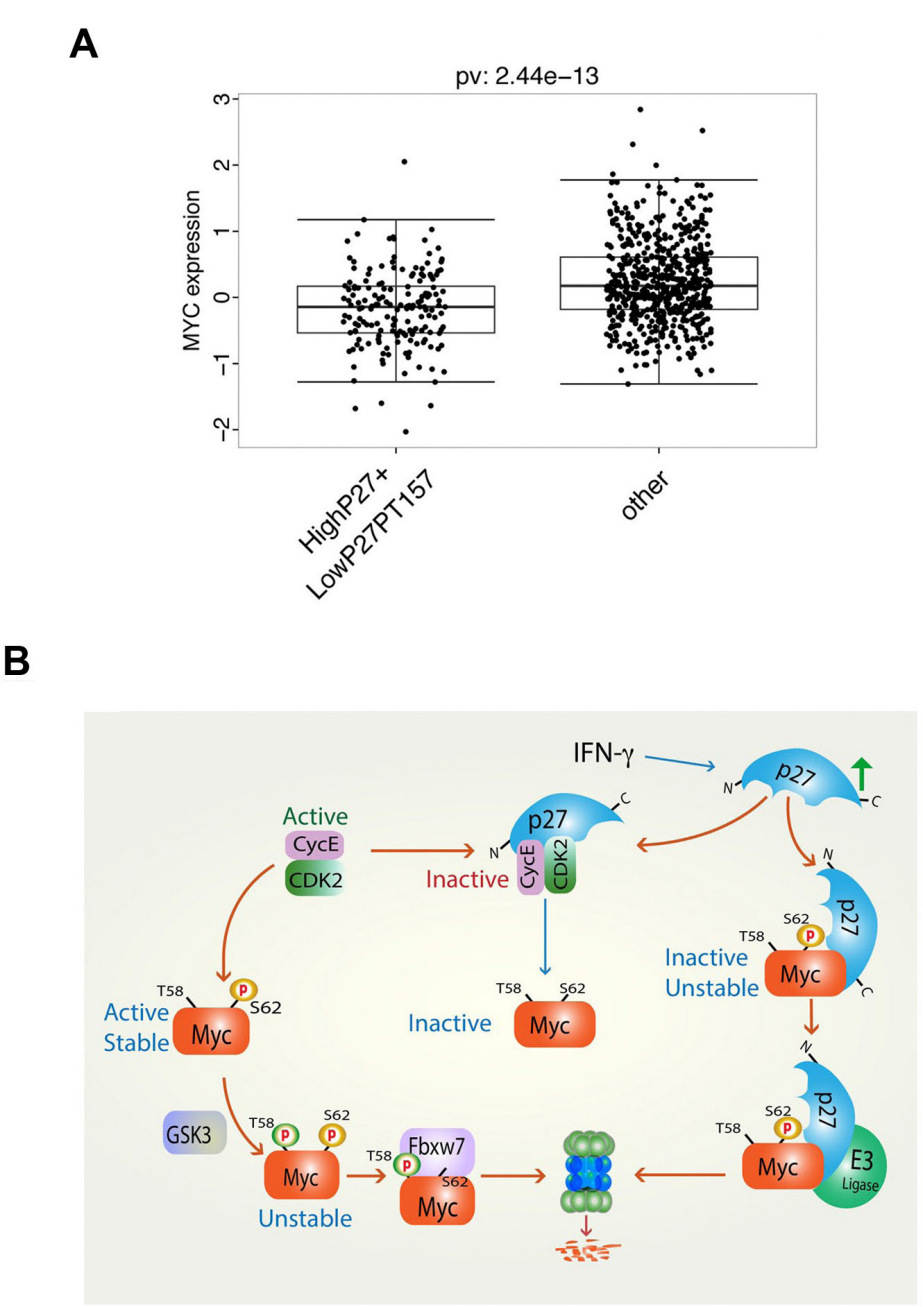
High level of total p27 protein expression together with low level of p27 Thr-157 phosphorylation correlates with low level of Myc protein expression in human breast cancer (**A**) Analysis of reverse phase protein array (RPPA) protein expression data for breast invasive carcinoma (BRCA) from The Cancer Genome Atlas (TCGA) containing data for 747 tumor samples. Correlation of Myc expression in a population with high p27 and low p27 phospho-Thr-157 vs. the other population. (**B**) Proposed model of regulation of Myc turnover and activity by IFN-γ, p27 and Cdk2. Left arm, active cyclin E/Cdk2 phosphorylates Myc at Ser-62 leading to an active, stable protein. Following GSK3β-mediated phosphorylation of Thr-58 primed by phospho-Ser-62, Myc is ubiquitylated by SCFF^bxw7^ and degraded by the proteasome. Middle arm, IFN-γ induces p27, which inhibits Cdk2 leading to reduced Ser-62 phosphorylation and inactivation of Myc. Right arm, IFN-γ-induced p27 binds Myc via its C-terminus leading to inactivation and degradation of Myc by an unidentified E3 ligase.

We next addressed the question whether high p27 expression in human tumors correlated with low Myc protein levels, which could be predicted from cell culture experiments, and whether this was linked to tumor grade and patient outcome. Myc and p27 protein expression data was obtained from The Cancer Genome Atlas (TCGA) data portal. The TCGA breast invasive carcinoma (BRCA) data set [36] contains protein expression data for 747 tumor samples measured by reverse phase protein array (RPPA) technology, and was thus of appropriate size for statistical analysis, while most other tumor data sets did not reach sufficient statistical power. Correlation analysis between MYC and p27 expression levels did not show significant correlation in the data set as a whole (Supplementary Figure S4A, S4B). Since high expression of cytoplasmic p27, which would not interfere with Cdk2 or the nuclear functions of Myc, occurs in many tumors as a result of phosphorylation of p27 by Akt, Rsk or Pim1, we selected a subpopulation with high total level of p27 expression but low level of phosphorylation at the T157 Akt/Rsk/Pim1 phosphorylation site (Supplementary Figure S4B). As shown in Figure 7A, Myc protein levels were significantly reduced in this subset of tumors (*p* = 2.44e−13), and a strong correlation (r = 0.5) between Myc protein level and p27 T157 phosphorylation was observed (Supplementary Figure S4A).

We next correlated the selected group of breast cancer patients with high p27 expression, low p27 T157 phosphorylation and low Myc protein level to clinical parameters (high and low meaning above or below median, respectively). Supplementary Table S1 shows that the selected population correlated positively with grade I tumors, luminal A subtype, estrogen receptor (ER) positivity and Her2 negativity (which are all favorable prognostic markers) and negatively correlated with grade III and IV tumors, basal and Her2 subtypes, ER negativity and Her2 positivity (which are all bad prognostic markers). Since the retinoblastoma protein (pRb) is an important target of Cdk2 and other Cdks, another functional criterion for p27 activity towards Cdks is the level of pRb phosphorylation. In agreement with this, we found that high p27 expression correlated with reduced pRb phosphorylation at Cdk-sites in the TCGA breast cancer RPPA data set (Supplementary Figure S4A). We therefore chose to select a population with high p27 expression, low Myc protein level and low pRb phosphorylation. The positive and negative correlations to tumor grade, molecular subtype and ER status were very similar to the previously selected group (Supplementary Table S2). Selecting patients with high p27 and low Myc protein levels without any other criteria also correlated to these clinical parameters, although less pronounced compared to the other two selected groups, while correlations to Myc or p27 alone had weaker effects (Supplementary Tables S3–S5). Notably, the selected group with high p27 expression, low Myc protein level and low pRB phosphorylation correlated significantly with relapse-free patient survival (*p* = 4.74e–02), while we observed the same trend for overall patient survival (Supplementary Figure S5). The groups with p27 expression together with low Myc protein level and high p27 expression alone also correlated with favorable outcome albeit less pronounced, while low Myc protein level alone actually displayed non-favorable overall survival, although this did not reach significance (Supplementary Figure S5). Unfortunately the population with high p27 expression, low p27 T157 phosphorylation and low Myc protein level was too small to obtain enough statistical power for this analysis (data not shown).

## DISCUSSION

The activity and stability of the oncoprotein Myc is controlled by posttranslational modifications and protein-protein interactions [1–5, 37]. We have reported previously that phosphorylation of Myc at Ser-62 by cyclin E/Cdk2 activates Myc function with respect to suppression of cellular senescence, and that this function in turn is reversed by expression of the Cdk2 inhibitor p27 and by pharmacological Cdk2 inhibitors [15]. Here we show that p27 overrides Myc's suppression of senescence independent on Ser-62 phosphorylation and induces ubiquitin/proteasome-mediated degradation of Myc (Figure 1). Further, IFN-γ, which restores differentiation and senescence in tumor cells overexpressing Myc, induces expression of p27 and promotes degradation of Myc in a p27-dependent manner (Figure 2). IFN-γ/p27-induced degradation of Myc is independent of Thr-58 phosphorylation and the E3 ligases SCF^Fbxw7^ and of SCF^Skp2^, which have been connected to Myc turnover previously, the former through MB1 and the latter connected to p27 [12, 19, 20, 23, 24]. This implicates an as yet unidentified E3 ligase in Myc regulation (see proposed model, Figure 7B). The identification of this E3 ligase will be an important task for the future.

Cdk2 is the best-known target for the anti-proliferative activity of p27. An important point of this work is that p27 not only affects Myc by binding to and inhibiting Cdk2, but also by interacting directly or indirectly with Myc (Figures 5 and 6), thereby identifying Myc as a new important target of p27. The interaction takes place directly at Myc target gene promoters and correlates with reduced Myc binding to chromatin and downregulated expression of these target genes (Figure 5A, 5B) [15]. The net outcome of p27 binding to Myc might therefore result in both reduced Myc activity and increased Myc turnover. In support of a role of p27 in gene transcription, two recent studies demonstrated regulation of transcription by p27 through interaction with p130/E2F4 and with the estrogen receptor α [38, 39].

Binding of Myc to p27 was shown to be dependent on the presence of amino acids 294–366 of Myc, which includes MB4, the NLS and the basic DNA-binding region. This region contains several ubiquitylation/acetylation sites implicated both in positive and negative regulation of Myc [21, 22, 37, 40, 41]. Some of these sites are targeted by the E3 ligases Huwe1/HectH9 and Fbxo28, leading to enhanced Myc activity by increasing the association with acetyl transferase/coactivator p300 [21, 22]. Further, the deacetylase Sirt1, which stabilizes and enhances the activity of Myc, is reported to target this region [40]. Interestingly, Fbxo28 activity is positively regulated by Cdk2 [22], and silencing of Sirt1, which is a negative regulator of p27, reduces lung tumor cell growth by inducing senescence in a p27-dependent manner [42]. MB4 has been reported to be involved in DNA binding, apoptosis, transformation and G2 arrest [43]. Any possible crosstalk between p27 and other factors binding to this region of Myc remains to be elucidated in the future.

The binding of p27 to Myc required the C-terminal half of p27, but not the N-terminus that binds to and inhibits Cdk2. The C-terminal region contains a number of regulatory phosphorylation sites and is involved in p27 protein stability, nuclear localization, protein interactions as well as CDK-independent functions of p27 in the cytoplasm [26, 28, 44, 45]. For instance, phosphorylation at Thr-187 by Cdk2 induces ubiquitin/proteasome-mediated turnover of p27 through interaction with the E3 ligase SCF^Skp2^ [26–28]. The Akt, Rsk and Pim1 kinases phosphorylate different sites in the C-terminus of p27 that regulates nuclear export [26–28]. Cytoplasmic p27 has been reported to exhibit both oncogenic and anti-oncogenic functions through interacting with RhoA and Rac1, thereby regulating cell migration and invasion [26, 28, 44, 45]. However, the interaction between Myc and p27 was found localized in the nucleus as demonstrated by BiFC and ChIP (Figure 5), arguing against nuclear export of Myc as a mechanism for degradation/inactivation of Myc through p27. The interaction between the C-terminal part of p27 and Myc therefore need to be characterized further in the future.

Interestingly, C-terminal part of p27 was sufficient to induce Myc degradation. It is at present unclear by what mechanism the C-terminus regulates Myc turnover. It is conceivable that the binding of p27 to Myc either involves recruitment of an E3 ligase that degrades Myc or alternatively excludes binding of a protein that stabilizes Myc, but at present we do not have any evidence that the binding of the C-terminus p27 to Myc is required for Myc degradation or whether this fragment acts through some other mechanism.

Myc and p27 is thus involved in a mutually antagonistic relation to each other. It has been shown previously that Myc antagonizes p27. Myc directly represses transcription from the p27 promoter [46] and inhibits p27 activity by inducing expression of cyclin D2, which sequesters p27. Further, Myc stimulates p27 degradation by promoting Cdk2-mediated phosphorylation of p27 at Thr-187 and by inducing expression of Skp2 and other components of the SCFSkp2 E3 ligase complex that recognizes this phosphorylation. We show here that p27 in turn antagonizes Myc upon anti-proliferative signaling. This is consistent with the opposite biological functions of p27 and Myc in regulation of the cell cycle, stem cell function, quiescence and senescence [1–5, 26–28], and previous observations that Mxd1, a Myc antagonist, cooperates with p27 to promote granulocytic differentiation [47] and that loss of p27 synergizes with Myc in murine lymphomagenesis [48, 49].

High Myc copy number/expression in tumors is known to correlate with high tumor grade and poor prognosis in a number of different tumors [1–5, 26–28]. Reduced p27 protein expression is frequently observed in human tumors such as breast, colorectal, prostate, head and neck, lung and gastric cancers, leukemia, lymphoma, neuroblastoma and melanoma, and is often a bad prognostic factor in these diseases [26–28]. This often goes together with high Skp2 expression in these tumors. Cytoplasmic relocalization of p27 occurs in response to Akt signaling and other oncogenic stimuli, thereby rendering it unable to target Cdk2 and Myc, and redirect p27 function to regulate RhoA, Rac and cell motility and is generally associated with poor prognosis, high tumor grade or metastasis in several human malignancies [26, 50–3]. Other reported mechanisms of down-regulating p27 expression or tumor suppressor activity in tumors include mutations, repression of p27 transcription or translation of p27 mRNA [26–28]. On the other hand, p27 is highly expressed in for instance normal breast epithelium and hyperplasia but is downregulated in ductal breast carcinoma *in situ* and invasive breast cancer [54]. High p27 expression in response to hormone treatment in breast cancer, to radiation in cervical and laryngeal cancer and to chemotherapy in small cell lung cancer is a good prognostic factor [26, 55]. However, in some other cancers, such as ovarian cancer, low p27 expression is better predictive marker for therapy, likely because rapidly dividing cells are more vulnerable to such treatments. We investigated the correlation between Myc and p27 protein levels in relation to tumor grade and patient outcome by analyzing reversed phase protein array (RPPA) data obtained from the TCGA breast invasive carcinoma (BRCA) data set, which has enough statistical power for this type of analysis. Although p27 and Myc protein levels did not correlate in the data set as a whole, we found a significant downregulation of Myc protein level in a subset of tumors with high p27 expression and low phosphorylation at the Thr-157 Akt/Rsk/Pim1 phosphorylation site. Phosphorylation at this site, which directs p27 nuclear export and thus renders p27 unable to inhibit Cdk2 and to interact with Myc in the nucleus, correlated significantly with increased Myc protein level. The selected population with high p27 expression, low Myc protein level and low Thr-157 phosphorylation correlated positively with grade I tumors, luminal A subtype, estrogen receptor (ER) positivity and Her2 negativity, while correlating negatively with grade III and IV tumors, basal and Her2 subtypes, ER negativity and Her2 positivity, which are all favorable and non-favorable prognostic markers, respectively. Very similar clinical results were obtained after selection of a population with high p27 expression, low Myc protein level and low phosphorylation of the retinoblastoma protein (pRb) at Cdk-sites, which was used as a functional readout of p27 activity. Notably, high p27 expression, low Myc protein level and low pRB phosphorylation correlated significantly with relapse-free patient survival, and the same trend was observed for overall patient survival (Supplementary Figure S5). High p27 expression together with low Myc protein level or high p27 expression alone also correlated with favorable outcome although less pronounced, while low Myc protein level alone did not. The statistical analysis is limited by the small size of RPPA data sets in breast cancer and is even more limited for other tumors types. It will therefore be of interest to redo this analysis in the future for different types of tumors when more extensive RPPA and other protein expression data are available.

Although we cannot say whether there is a causal relationship between high p27 and low Myc protein in these tumors, this would be compatible with a scenario where high p27 expression causes downregulation of Myc protein levels. Inverse relation between p27 and Myc protein levels has previously been observed in tumor biopsies from chronic lymphocytic leukemia (CLL) [56] as well as after *H. pylori* eradication in chronic gastritis [57]. It was recently reported that a high Myc/high phospho-Rb/low p27 signature was a poor prognostic marker in breast and ovarian cancer [58], which is compatible with our data and again emphasizes the inverse relationship between Myc and p27. As larger protein expression data sets for various tumors will be available in the future it will be interesting to expand these studies to other tumor types.

In summary, we show here that p27 targets Myc both indirectly through Cdk2 and directly by binding Myc (see proposed model, Figure 7B). The biological consequences of this is increased Myc turnover, loss of Myc from target gene promoters, reduced expression of Myc target genes, induction of growth arrest, senescence and differentiation of Myc-driven tumor cells. Further, we show that Myc and p27 exhibit an inverse relationship in many tumors that is of prognostic value. Finding means to enforce p27 expression/activity could therefore be a new way of combating Myc. Treatments utilizing pharmacological CDK2 inhibitors, which are already in clinical development, is one approach to mimic part of the anti-Myc function of p27. Other approaches to enforce p27 that is ongoing is inhibition of Skp2 [59–62] and inhibition of kinases that promote nuclear export of p27 [63]. Cytokines like IFN-γ and TGF-β, as well as differentiation, cell density and adhesion signals that upregulate p27 [26, 28] have been shown to inhibit growth of Myc–driven tumor cells in different systems including the one described here [15, 47, 64]. IFN-γ is produced by CD4+ Th1 T-lymphocytes and plays an important role in immunosurveillance of tumors by activating cytotoxic T-cells that eliminates tumor cells [65]. However, recent studies suggest that IFN-γ and other cytokines produced by CD4+ Th1 T-cells also targets tumor cells directly *in vivo* by inducing cellular senescence [66] thereby keeping tumors in check, which is consistent with our findings. Immunotherapeutic approaches supporting IFN-γ-producing T cells infiltrating Myc-driven tumors could therefore be a plausible complementary approach to molecular therapies targeting Cdk2 or signaling pathways enhancing p27 expression/activity.

## MATERIALS AND METHODS

### Cell culture, differentiation and senescence assays

Cells were cultured in RPMI-1640 medium (U-937, 2fTGH, Colo-320, Fbxw7/Cdc4^−/−^HCT116) or Dulbecco's modified essential medium (DMEM) (Cos 7, HeLa, and U2OS) supplemented with 10% fetal calf serum and antibiotics. The U937 clone myc-6 expresses the OK10 v-*myc* gene [29]. Exponentially growing U-937 cells (10^5^/mL) and Colo-320 cells were treated with 1.6 × 10^8^ mol/L TPA (Sigma, St Louis, MO) and/or 100 U/mL IFN-γ (1000 U/ml for 2fTGH cells) (generously provided by Dr. G. R. Adolf, Ernst-Boehringer Institute, Vienna, Austria). ^3^H-thymidine incorporation assays and FACS analysis of CD11c expression were performed as described earlier [67]. Analysis of senescence by SA-β-Gal staining was performed as described [15].

### Transfections and plasmids

For gene transfer, subconfluent cells were transfected using FuGENE6 (Boehringer Mannheim), Lipofectamine (BD Biosciences) or Superfect (Qiagen) according to manufacturer's instructions, or were electroporated as described [67]. The following plasmids were used in transfections: CMV-Myc, CMV-MycT58A, pCGN-HA-Myc, pCGN-HA-Myc95–439 (ΔN1), 148–439 (ΔN2), 221–439 (ΔN3), 294–439 (ΔN4), 367–439 (ΔN5), pCMV-p27, pCMV-p27aa1-87 (N100), pCMV-p27aa82–198 (N101).

### Protein, mRNA and ChIP assays

Pulse chase, immunoprecipitation and immunoblot analyses were performed as described previously [24, 67]. The following antibodies were used in these assays: α-c-Myc (C33), α-c-Myc (N262), α-c-Myc (9E10), α-Max (C-17), α-p27 (C-19), α-p27 (F-8), α-Gal4 (all from Santa Cruz Biotechnology Inc. (SCB, Santa Cruz, CA, USA)), α-p27 (Kip1) (BD Biosciences), α-Flag M2 (Sigma), α-HA-3F10 (Roche), α-Ub clone FK2 (Affiniti), and IG-C rabbit pan-Myc antiserum.

The proteasome inhibitor Z-leu-leu-leu-H aldehyde (MG115) (Peptides International) and N-acetyl-leucinyl-leucinyl-norleucinal-H (LLnL) (Sigma) or vehicle were added to the cells 2 hrs before harvest at a concentration of 50 μM. To block protein synthesis, cycloheximide (CHX) was applied at a concentration of 100 μg/ml.

Chromatin immunoprecipitations were performed as detailed [24]. Briefly, cells were crosslinked with 1% formaldehyde on ice for 6 minutes. Nuclear chromatin was sonicated on ice to fragments from 0.3 kb to 0.5 kb. Nuclear chromatin equivalent to 2.5 × 10^7^ cells was immunoprecipitated with 2 μg antibody. The following antibodies were used in CHIP: α-pan-Myc (IG-C), and pre-immune serum (IG-0). Primer sequences for the *cyclin* D2 promoter (E-box binding site region) used in Quantitative real-time PCR: forward primer 5′ CCCCTTCCTCCTGGAGTGAAATAC, reverse primer 5′ CGTGCTCTAACGCATCCTTGA-GTC. RT-qPCR analysis of mRNA expression of CCND2 and MYC was performed as described [24]. Primer sequences CCND2: Forward primer: 5′GCTGGCTAAGATCACCAACACA, reverse primer: 5′GCACCGCCTCAATCTGC. Human MYC: Forward primer: 5′TTCGGGTAGTGGAAAACCAG, reverse primer: 5′AGTAGAAATACGGCTGCACC. Mouse MYC: Forward primer: 5′GCTGTTTGAAGGCTGGATTTC, reverse primer: 5′GATGAAATAGGGCTGTACGGAG.

### Fluorescence microscopy

2fTGH cells were cotransfected with constructs containing Myc-GFP together with CFP. BiFC was performed essentially as described [24] using constructs containing c-Myc fused to the C-terminal fragment of YFP (MycYC), Max fused to the N-terminal fragment of YFP (MaxYN) or ubiquitin fused to the N-terminal fragment of YFP (YNUb) (kindly provided by T. Kerppola, Ann Arbor, Michigan). Fluorescence emissions were observed in living cells using an inverted microscope (Zeiss Axiovert 200 M, Goettingen, Germany) together with software from Improvision (OpenLab 4.0.1), and black and white images were captured with a Hamamatsu ORCA-ER digital camera, where non-treated cells were used as reference for exposure settings. CFP, GFP and YFP fluorescence was measured by excitation at 436, 470 and 513 nm, respectively, and emission at 470, 505 and 535 nm, respectively. Quantification of fluorescence intensities was performed via software from Improvision (Volocity 3.0), on images captured and processed as described above.

### Statistical analysis of RPPA protein expression data

Protein expression data for breast invasive carcinoma (BRCA) from The Cancer Genome Atlas (TCGA) [36] were accessed via the UCSC Cancer Browser [68]. Specifically, the data set contains protein expression data for 747 tumor samples measured by the reverse phase protein array (RPPA) technology. For subsequent analyses of patient groups showing either high or low expression levels of one or more of the indicated target proteins, patients were split into low and high expression groups based on the median expression of the respective proteins.

All analyses were performed in R (version 3.1.1). Associations between the expression levels of the target proteins were evaluated using Spearman's rank correlation coefficient. The correlations were derived using the rcorr function from the Hmisc package. Differences in Myc protein levels between patients with high p27 expression but low expression levels of p27PT157 and the remaining patient samples were analyzed using the Wilcoxon rank sum test from the R stats package. Differences in survival time between the indicated patient groups were assessed using the R survival package. A significance level of 5% was considered as statistically significant.

## SUPPLEMENTARY MATERIALS FIGURES AND TABLES



## References

[1] Dang CV (2012). Myc on the path to cancer. Cell.

[2] Eilers M, Eisenman RN (2008). Myc's broad reach. Genes Dev.

[3] Larsson LG, Henriksson MA (2010). The yin and yang functions of the myc oncoprotein in cancer development and as targets for therapy. Exp Cell Res.

[4] Luscher B, Vervoorts J (2012). Regulation of gene transcription by the oncoprotein myc. Gene.

[5] Meyer N, Penn LZ (2008). Reflecting on 25 years with myc. Nat Rev Cancer.

[6] Lin CY, Loven J, Rahl PB, Paranal RM, Burge CB, Bradner JE, Lee TI, Young RA (2012). Transcriptional amplification in tumor cells with elevated c-myc. Cell.

[7] Nie Z, Hu G, Wei G, Cui K, Yamane A, Resch W, Wang R, Green DR, Tessarollo L, Casellas R, Zhao K, Levens D (2012). c-Myc is a universal amplifier of expressed genes in lymphocytes and embryonic stem cells. Cell.

[8] Sabo A, Kress TR, Pelizzola M, de Pretis S, Gorski MM, Tesi A, Morelli MJ, Bora P, Doni M, Verrecchia A, Tonelli C, Faga G, Bianchi V (2014). Selective transcriptional regulation by Myc in cellular growth control and lymphomagenesis. Nature.

[9] Walz S, Lorenzin F, Morton J, Wiese KE, von Eyss B, Herold S, Rycak L, Dumay-Odelot H, Karim S, Bartkuhn M, Roels F, Wustefeld T, Fischer M (2014). Activation and repression by oncogenic MYC shape tumour-specific gene expression profiles. Nature.

[10] Herkert B, Eilers M (2010). Transcriptional repression: The dark side of myc. Genes Cancer.

[11] Beroukhim R, Mermel CH, Porter D, Wei G, Raychaudhuri S, Donovan J, Barretina J, Boehm JS, Dobson J, Urashima M, Mc Henry KT, Pinchback RM, Ligon AH (2010). The landscape of somatic copy-number alteration across human cancers. Nature.

[12] Farrell AS, Sears RC (2014). Myc degradation. Cold Spring Harb Perspect Med.

[13] Bahram F, von der Lehr N, Cetinkaya C, Larsson LG (2000). C-myc hot spot mutations in lymphomas result in inefficient ubiquitination and decreased proteasome-mediated turnover. Blood.

[14] Salghetti SE, Kim SY, Tansey WP (1999). Destruction of myc by ubiquitin-mediated proteolysis: Cancer-associated and transforming mutations stabilize myc. Embo J.

[15] Hydbring P, Bahram F, Su Y, Tronnersjo S, Hogstrand K, von der Lehr N, Sharifi HR, Lilischkis R, Hein N, Wu S, Vervoorts J, Henriksson M, Grandien A (2010). Phosphorylation by Cdk2 is required for Myc to repress Ras-induced senescence in cotransformation. Proceedings of the National Academy of Sciences of the United States of America.

[16] Lutterbach B, Hann SR (1994). Hierarchical phosphorylation at n-terminal transformation-sensitive sites in c-myc protein is regulated by mitogens and in mitosis. Mol Cell Biol.

[17] Sears R, Nuckolls F, Haura E, Taya Y, Tamai K, Nevins JR (2000). Multiple ras-dependent phosphorylation pathways regulate myc protein stability. Genes Dev.

[18] Yeh E, Cunningham M, Arnold H, Chasse D, Monteith T, Ivaldi G, Hahn WC, Stukenberg PT, Shenolikar S, Uchida T, Counter CM, Nevins JR, Means AR (2004). A signalling pathway controlling c-Myc degradation that impacts oncogenic transformation of human cells. Nature cell biology.

[19] Welcker M, Orian A, Jin J, Grim JE, Harper JW, Eisenman RN, Clurman BE (2004). The fbw7 tumor suppressor regulates glycogen synthase kinase 3 phosphorylation-dependent c-myc protein degradation. Proc Natl Acad Sci USA.

[20] Yada M, Hatakeyama S, Kamura T, Nishiyama M, Tsunematsu R, Imaki H, Ishida N, Okumura F, Nakayama K, Nakayama KI (2004). Phosphorylation-dependent degradation of c-myc is mediated by the f-box protein fbw7. Embo J.

[21] Adhikary S, Marinoni F, Hock A, Hulleman E, Popov N, Beier R, Bernard S, Quarto M, Capra M, Goettig S, Kogel U, Scheffner M, Helin K (2005). The ubiquitin ligase HectH9 regulates transcriptional activation by Myc and is essential for tumor cell proliferation. Cell.

[22] Cepeda D, Ng HF, Sharifi HR, Mahmoudi S, Cerrato VS, Fredlund E, Magnusson K, Nilsson H, Malyukova A, Rantala J, Klevebring D, Vinals F, Bhaskaran N (2013). CDK-mediated activation of the SCF(FBXO) (28) ubiquitin ligase promotes MYC-driven transcription and tumourigenesis and predicts poor survival in breast cancer. EMBO molecular medicine.

[23] Kim SY, Herbst A, Tworkowski KA, Salghetti SE, Tansey WP (2003). Skp2 regulates myc protein stability and activity. Mol Cell.

[24] von der Lehr N, Johansson S, Wu S, Bahram F, Castell A, Cetinkaya C, Hydbring P, Weidung I, Nakayama K, Nakayama KI, Soderberg O, Kerppola TK, Larsson LG (2003). The F-box protein Skp2 participates in c-Myc proteosomal degradation and acts as a cofactor for c-Myc-regulated transcription. Molecular cell.

[25] Hydbring P, Larsson LG (2010). Tipping the balance: Cdk2 enables myc to suppress senescence. Cancer Res.

[26] Chu IM, Hengst L, Slingerland JM (2008). The cdk inhibitor p27 in human cancer: Prognostic potential and relevance to anticancer therapy. Nature reviews. Cancer.

[27] Lu Z, Hunter T (2010). Ubiquitylation and proteasomal degradation of the p21(cip1), p27(kip1) and p57(kip2) cdk inhibitors. Cell Cycle.

[28] Vervoorts J, Luscher B (2008). Post-translational regulation of the tumor suppressor p27(kip1). Cell Mol Life Sci.

[29] Oberg F, Larsson LG, Anton R, Nilsson K (1991). Interferon gamma abrogates the differentiation block in v-myc-expressing u-937 monoblasts. Proc Natl Acad Sci USA.

[30] Tworkowski KA, Salghetti SE, Tansey WP (2002). Stable and unstable pools of myc protein exist in human cells. Oncogene.

[31] Larsson LG, Pettersson M, Oberg F, Nilsson K, Luscher B (1994). Expression of mad, mxi1, max and c-myc during induced differentiation of hematopoietic cells: Opposite regulation of mad and c-myc. Oncogene.

[32] Hu CD, Chinenov Y, Kerppola TK (2002). Visualization of interactions among bzip and rel family proteins in living cells using bimolecular fluorescence complementation. Mol Cell.

[33] Arabi A, Wu S, Ridderstrale K, Bierhoff H, Shiue C, Fatyol K, Fahlen S, Hydbring P, Soderberg O, Grummt I, Larsson LG, Wright AP (2005). c-Myc associates with ribosomal DNA and activates RNA polymerase I transcription. Nature cell biology.

[34] Ramana CV, Gil MP, Schreiber RD, Stark GR (2002). Stat1-dependent and -independent pathways in ifn-gamma-dependent signaling. Trends Immunol.

[35] Salghetti SE, Kim SY, Tansey WP (1999). Destruction of myc by ubiquitin-mediated proteolysis: Cancer-associated and transforming mutations stabilize myc. Embo J.

[36] (2012). Comprehensive molecular portraits of human breast tumours. Nature.

[37] Vervoorts J, Luscher-Firzlaff J, Luscher B (2006). The ins and outs of myc regulation by posttranslational mechanisms. J Biol Chem.

[38] Pippa R, Espinosa L, Gundem G, Garcia-Escudero R, Dominguez A, Orlando S, Gallastegui E, Saiz C, Besson A, Pujol MJ, Lopez-Bigas N, Paramio JM, Bigas A (2012). p27Kip1 represses transcription by direct interaction with p130/E2F4 at the promoters of target genes. Oncogene.

[39] Jeon MJ, Yang W, Seo HS, Wang ES, Shin I (2012). Down-regulation of estrogen receptor alpha (eralpha) transcriptional activity by p27 is mediated by inhibition of eralpha nuclear localization and modulation of the eralpha transcriptional complex. Cell Signal.

[40] Menssen A, Hydbring P, Kapelle K, Vervoorts J, Diebold J, Luscher B, Larsson LG, Hermeking H (2012). The c-myc oncoprotein, the nampt enzyme, the sirt1-inhibitor dbc1, and the sirt1 deacetylase form a positive feedback loop. Proc Natl Acad Sci USA.

[41] Wasylishen AR, Chan-Seng-Yue M, Bros C, Dingar D, Tu WB, Kalkat M, Chan PK, Mullen PJ, Huang L, Meyer N, Raught B, Boutros PC, Penn LZ (2013). MYC phosphorylation at novel regulatory regions suppresses transforming activity. Cancer research.

[42] Zhu L, Chiao CY, Enzer KG, Stankiewicz AJ, Faller DV, Dai Y (2015). Sirt1 inactivation evokes antitumor activities in nsclc through the tumor suppressor p27. Mol Cancer Res.

[43] Cowling VH, Chandriani S, Whitfield ML, Cole MD (2006). A conserved myc protein domain, mbiv, regulates DNA binding, apoptosis, transformation, and g2 arrest. Mol Cell Biol.

[44] Besson A, Gurian-West M, Schmidt A, Hall A, Roberts JM (2004). P27kip1 modulates cell migration through the regulation of rhoa activation. Genes Dev.

[45] McAllister SS, Becker-Hapak M, Pintucci G, Pagano M, Dowdy SF (2003). Novel p27(kip1) c-terminal scatter domain mediates rac-dependent cell migration independent of cell cycle arrest functions. Mol Cell Biol.

[46] Yang W, Shen J, Wu M, Arsura M, FitzGerald M, Suldan Z, Kim DW, Hofmann CS, Pianetti S, Romieu-Mourez R, Freedman LP, Sonenshein GE (2001). Repression of transcription of the p27(Kip1) cyclin-dependent kinase inhibitor gene by c-Myc. Oncogene.

[47] McArthur GA, Foley KP, Fero ML, Walkley CR, Deans AJ, Roberts JM, Eisenman RN (2002). Mad1 and p27(kip1) cooperate to promote terminal differentiation of granulocytes and to inhibit myc expression and cyclin e-cdk2 activity. Mol Cell Biol.

[48] Hwang HC, Martins CP, Bronkhorst Y, Randel E, Berns A, Fero M, Clurman BE (2002). Identification of oncogenes collaborating with p27kip1 loss by insertional mutagenesis and high-throughput insertion site analysis. Proc Natl Acad Sci USA.

[49] Martins CP, Berns A (2002). Loss of p27(kip1) but not p21(cip1) decreases survival and synergizes with myc in murine lymphomagenesis. Embo J.

[50] Liang J, Zubovitz J, Petrocelli T, Kotchetkov R, Connor MK, Han K, Lee JH, Ciarallo S, Catzavelos C, Beniston R, Franssen E, Slingerland JM (2002). PKB/Akt phosphorylates p27, impairs nuclear import of p27 and opposes p27-mediated G1 arrest. Nature medicine.

[51] Besson A, Assoian RK, Roberts JM (2004). Regulation of the cytoskeleton: An oncogenic function for cdk inhibitors? Nature reviews. Cancer.

[52] Besson A, Dowdy SF, Roberts JM (2008). Cdk inhibitors: Cell cycle regulators and beyond. Dev Cell.

[53] Denicourt C, Saenz CC, Datnow B, Cui XS, Dowdy SF (2007). Relocalized p27kip1 tumor suppressor functions as a cytoplasmic metastatic oncogene in melanoma. Cancer Res.

[54] Han S, Park K, Kim HY, Lee MS, Kim HJ, Kim YD (1999). Reduced expression of p27kip1 protein is associated with poor clinical outcome of breast cancer patients treated with systemic chemotherapy and is linked to cell proliferation and differentiation. Breast Cancer Res Treat.

[55] Oshita F, Kameda Y, Nishio K, Tanaka G, Yamada K, Nomura I, Nakayama H, Noda K (2000). Increased expression levels of cyclin-dependent kinase inhibitor p27 correlate with good responses to platinum-based chemotherapy in non-small cell lung cancer. Oncol Rep.

[56] Caraballo JM, Acosta JC, Cortes MA, Albajar M, Gomez-Casares MT, Batlle-Lopez A, Cuadrado MA, Onaindia A, Bretones G, Llorca J, Piris MA, Colomer D, Leon J (2014). High p27 protein levels in chronic lymphocytic leukemia are associated to low Myc and Skp2 expression, confer resistance to apoptosis and antagonize Myc effects on cell cycle. Oncotarget.

[57] Kim SS, Meitner P, Konkin TA, Cho YS, Resnick MB, Moss SF (2006). Altered expression of skp2, c-myc and p27 proteins but not mrna after h. Pylori eradication in chronic gastritis. Mod Pathol.

[58] Seviour EG, Sehgal V, Lu Y, Luo Z, Moss T, Zhang F, Hill SM, Liu W, Maiti SN, Cooper L, Azencot R, Lopez-Berestein G, Rodriguez-Aguayo C (2015). Functional proteomics identifies miRNAs to target a p27/Myc/phospho-Rb signature in breast and ovarian cancer. Oncogene.

[59] Rico-Bautista E, Yang CC, Lu L, Roth GP, Wolf DA (2010). Chemical genetics approach to restoring p27kip1 reveals novel compounds with antiproliferative activity in prostate cancer cells. BMC Biol.

[60] Wu L, Grigoryan AV, Li Y, Hao B, Pagano M, Cardozo TJ (2012). Specific small molecule inhibitors of skp2-mediated p27 degradation. Chem Biol.

[61] Ooi LC, Watanabe N, Futamura Y, Sulaiman SF, Darah I, Osada H (2013). Identification of small molecule inhibitors of p27(kip1) ubiquitination by high-throughput screening. Cancer Sci.

[62] Pavlides SC, Huang KT, Reid DA, Wu L, Blank SV, Mittal K, Guo L, Rothenberg E, Rueda B, Cardozo T, Gold LI (2013). Inhibitors of scf-skp2/cks1 e3 ligase block estrogen-induced growth stimulation and degradation of nuclear p27kip1: Therapeutic potential for endometrial cancer. Endocrinology.

[63] Morishita D, Takami M, Yoshikawa S, Katayama R, Sato S, Kukimoto-Niino M, Umehara T, Shirouzu M, Sekimizu K, Yokoyama S, Fujita N (2011). Cell-permeable carboxyl-terminal p27(kip1) peptide exhibits anti-tumor activity by inhibiting pim-1 kinase. J Biol Chem.

[64] Cetinkaya C, Hultquist A, Su Y, Wu S, Bahram F, Pahlman S, Guzhova I, Larsson LG (2007). Combined ifn-gamma and retinoic acid treatment targets the n-myc/max/mad1 network resulting in repression of n-myc target genes in mycn-amplified neuroblastoma cells. Mol Cancer Ther.

[65] Finn OJ (2008). Cancer immunology. N Engl J Med.

[66] Braumuller H, Wieder T, Brenner E, Assmann S, Hahn M, Alkhaled M, Schilbach K, Essmann F, Kneilling M, Griessinger C, Ranta F, Ullrich S, Mocikat R (2013). T-helper-1-cell cytokines drive cancer into senescence. Nature.

[67] Bahram F, Wu S, Oberg F, Luscher B, Larsson LG (1999). Posttranslational regulation of myc function in response to phorbol ester/interferon-gamma-induced differentiation of v-myc-transformed u-937 monoblasts. Blood.

[68] Zhu J, Sanborn JZ, Benz S, Szeto C, Hsu F, Kuhn RM, Karolchik D, Archie J, Lenburg ME, Esserman LJ, Kent WJ, Haussler D, Wang T (2009). The UCSC Cancer Genomics Browser. Nature methods.

