# Supplementary material for: Interferon-γ-induced p27KIP1 binds to and targets MYC for proteasome-mediated degradation
Source: Oncotarget. 2015 Dec 20;7(3):2837–54. doi: 10.18632/oncotarget.6693 (PMC4823075; doi:10.18632/oncotarget.6693)
Supplement: Supplementary file 1 [file oncotarget-07-2837-s001.pdf]

Identification of the BRAF V600E mutation in gastroenteropancreatic neuroendocrine tumors

Supplementary Materials

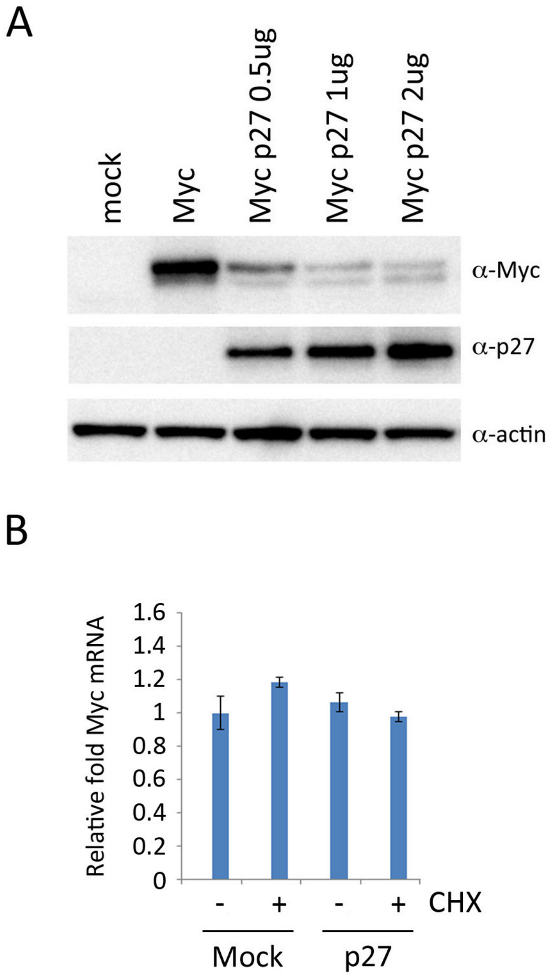

Supplementary Figure S1: p27 downregulates Myc protein levels in a dose-dependent manner independent of Myc mRNA levels. (**A**) HA-tagged c-Myc was cotransfected with p27 into HeLa cells at indicated concentrations. Western blot (WB) analysis of total cell lysates was carried out using HA antibodies (upper panel), p27 BD Ab (middle panel) and actin Ab (lower panel). (**B**) Empty vector (Mock) or p27 construct was transfected into HCT116−/− Fbxw7 cells. 48 hours post-transfection cells were treated with cycloheximide (CHX) for 2 hours, or left untreated, followed by harvest of total RNA. Human c-*Myc* mRNA levels were quantified through RT-qPCR, using the delta-delta-Ct-method, with GAPDH as reference gene.

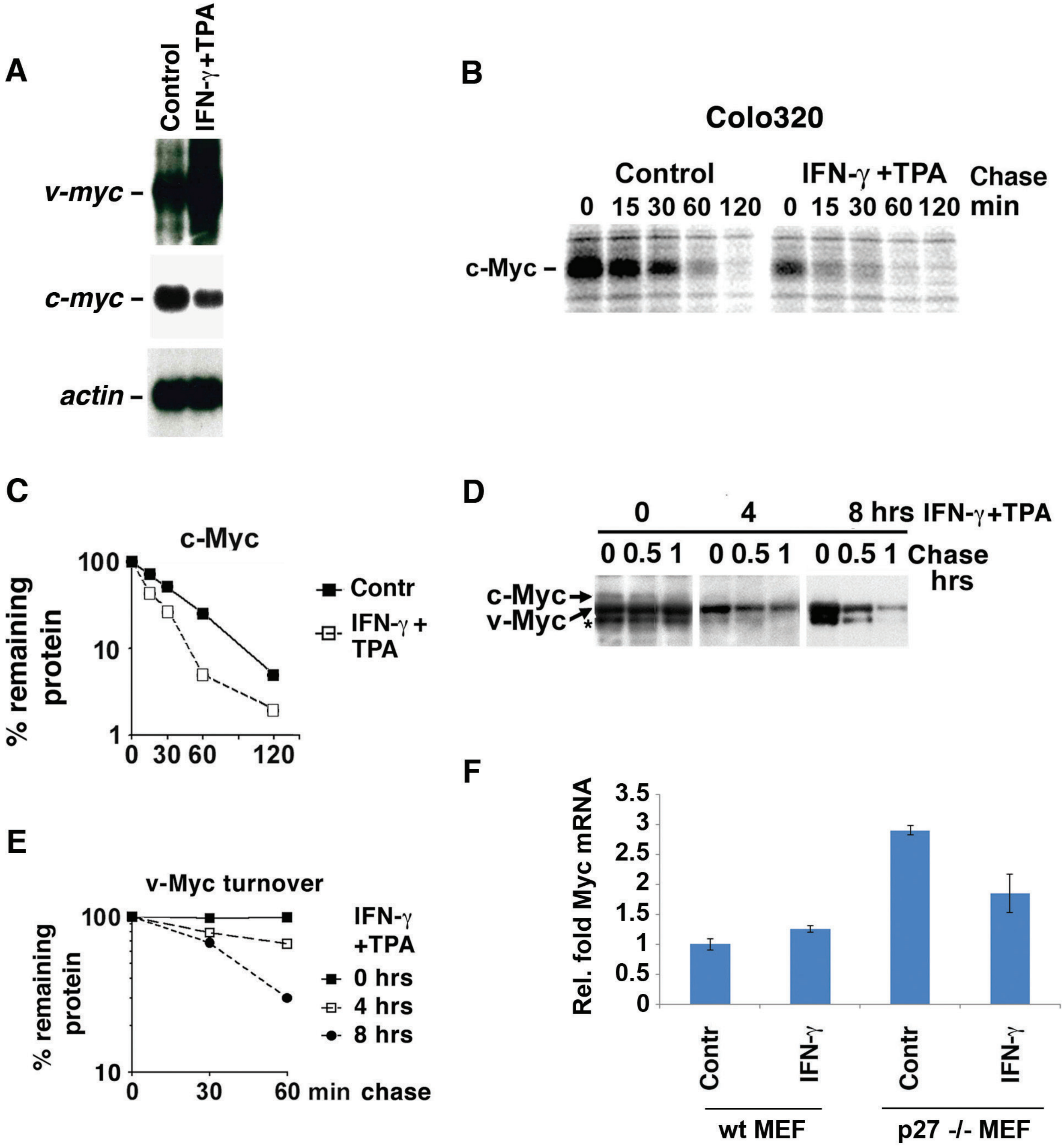

Supplementary Figure S2: IFN-γ+ TPA induces degradation of Myc in Colo-320 and U-937 cells. (**A**) Northern blot analysis of v- and c-Myc mRNA levels in U-937-myc-6 cells following treatment with IFN-γ+ TPA. (**B, C**) IFN-γ+ TPA treatment increases c-Myc degradation in Colo-320 colon carcinoma cells. The analysis was performed as described in the legend to Figure 2F. (**D, E**) Kinetics of induced Myc turnover as determined by CHX chase. (D) U-937-myc-6 cells were treated with IFN-γ+ TPA for 0, 4 or 8 hrs and CHX added for the indicated times. c-Myc was immunoprecipitated with Flag antibodies followed by western blot analysis using pan-Myc antibodies. Note that endogenous c-Myc expression is rapidly downregulated at the level of transcription after TPA (and IFN-γ+ TPA) treatment [31]. The lower band denoted "*" is seen occasionally after IFN-γ+ TPA treatment and may represent a degradation product of v-Myc. Note also that these samples were run on a minigel where c- and v-Myc migrates closer together than on the long 35S gels in Figure 2F. (E) Quantification was performed using a CCD camera. (**F**) Levels of c-Myc mRNA in mouse embryo fibroblasts (MEFs) in response to IFN-γ treatment. Wt and p27 −/− MEFs were treated with 5000 U of murine IFN-γ or vehicle, for 24 hours followed by harvest of total RNA. Quantification of murine c-Myc mRNA levels was performed as in Supplementary Figure 1B.

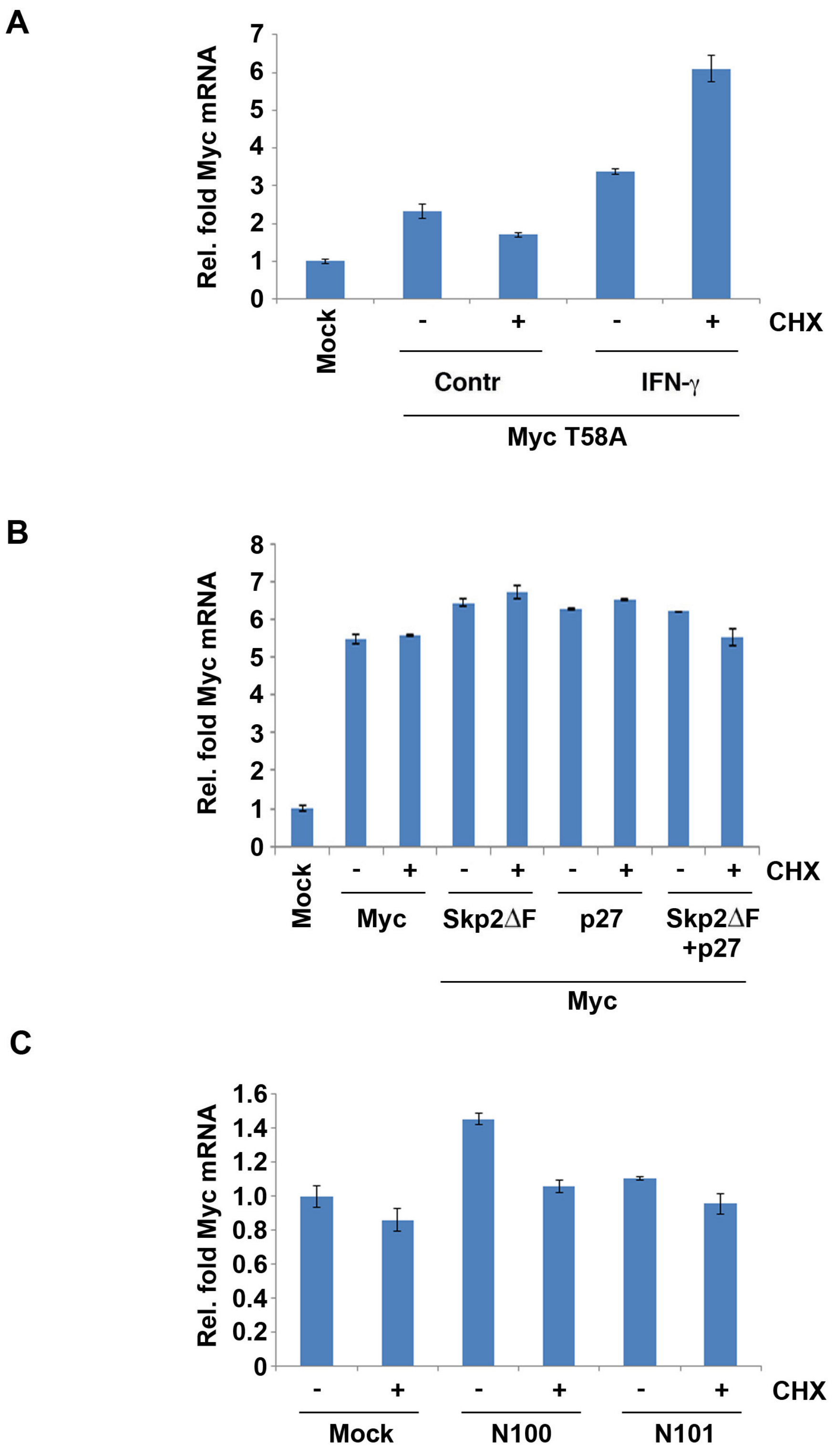

Supplementary Figure S3: Expression of c-Myc mRNA levels during IFN-γ treatment or ectopic expression of p27 constructs in 2fTGH, HeLa and HCT116-/-Fbxw7 cells. (**A**) Empty vector (Mock) or Myc-T58A construct was transfected into 2fTGH cells. 48 hours post-transfection cells were treated with 1000 U of human IFN-γ or vehicle, for 24 hours. 72 hours post-transfection cells were harvested for total RNA. Quantification of c-Myc mRNA levels was performed as in Supplementary Figure 1B. (**B**) Empty vector (Mock) or indicated expression constructs were transfected into HeLa cells. 48 hours post-transfection cells were treated with cycloheximide (CHX) for 2 hours, or left untreated, followed by harvest of total RNA. Quantification of c-Myc mRNA levels was performed as in Supplementary Figure 1B. (**C**) Empty vector (Mock), p27-deletion construct N100 or p27-deletion construct N101 was transfected into HCT116 −/− Fbxw7 cells. 48 hours post-transfection cells were treated with cycloheximide (CHX) for 2 hours, or left untreated, followed by harvest of total RNA. Quantification of c-Myc mRNA levels was performed as in Supplementary Figure 1B.

A

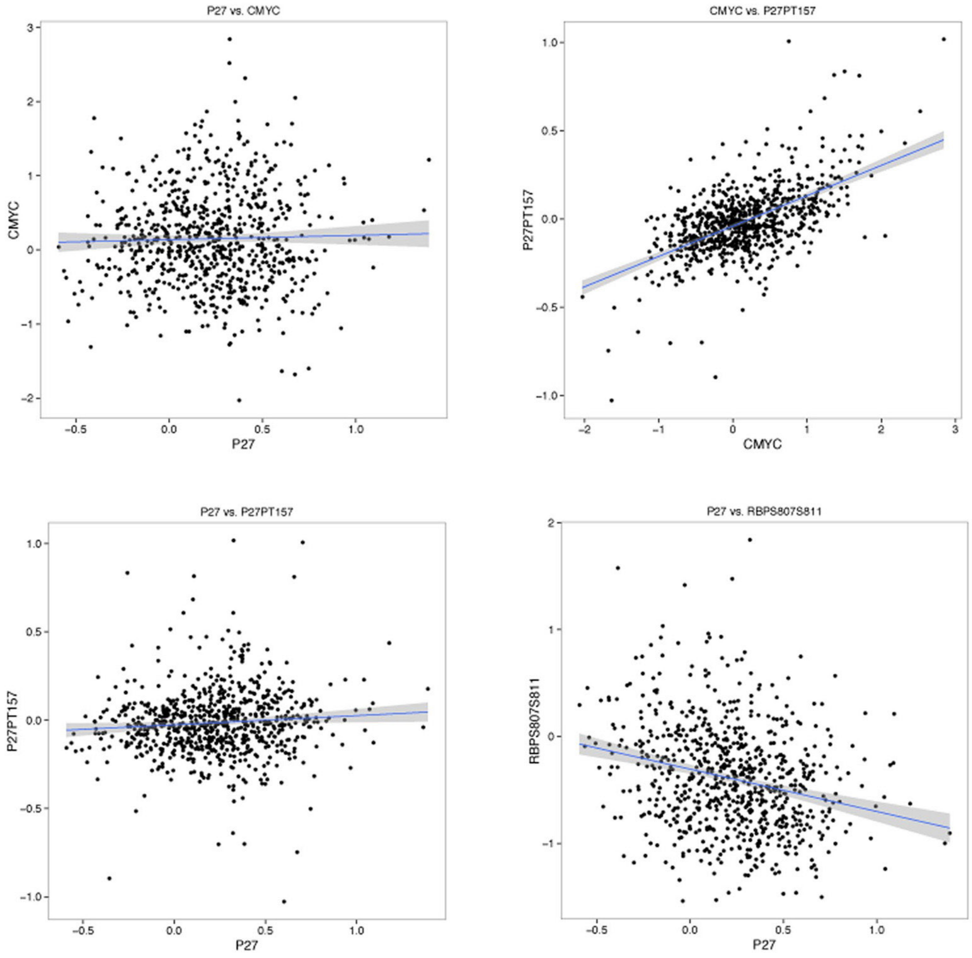

B

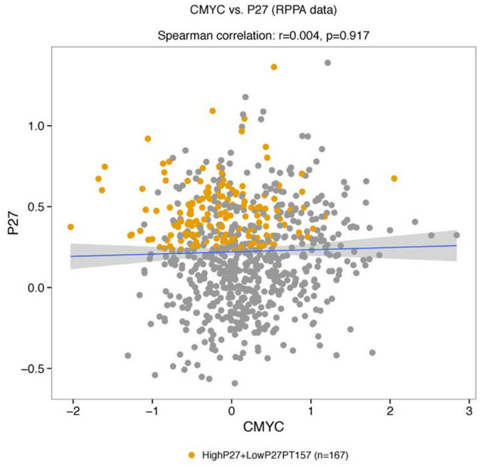

Supplementary Figure S4: Analysis of reverse phase protein array (RPPA) protein expression data for indicated proteins and phospho-proteins in breast invasive carcinoma (BRCA) from The Cancer Genome Atlas (TCGA). (**A**) Target correlation between indicated proteins/phosphoproteins. (**B**) Selection of a population with high p27, low p27 phospho-Thr-157 and low Myc protein expression highlighted in yellow.

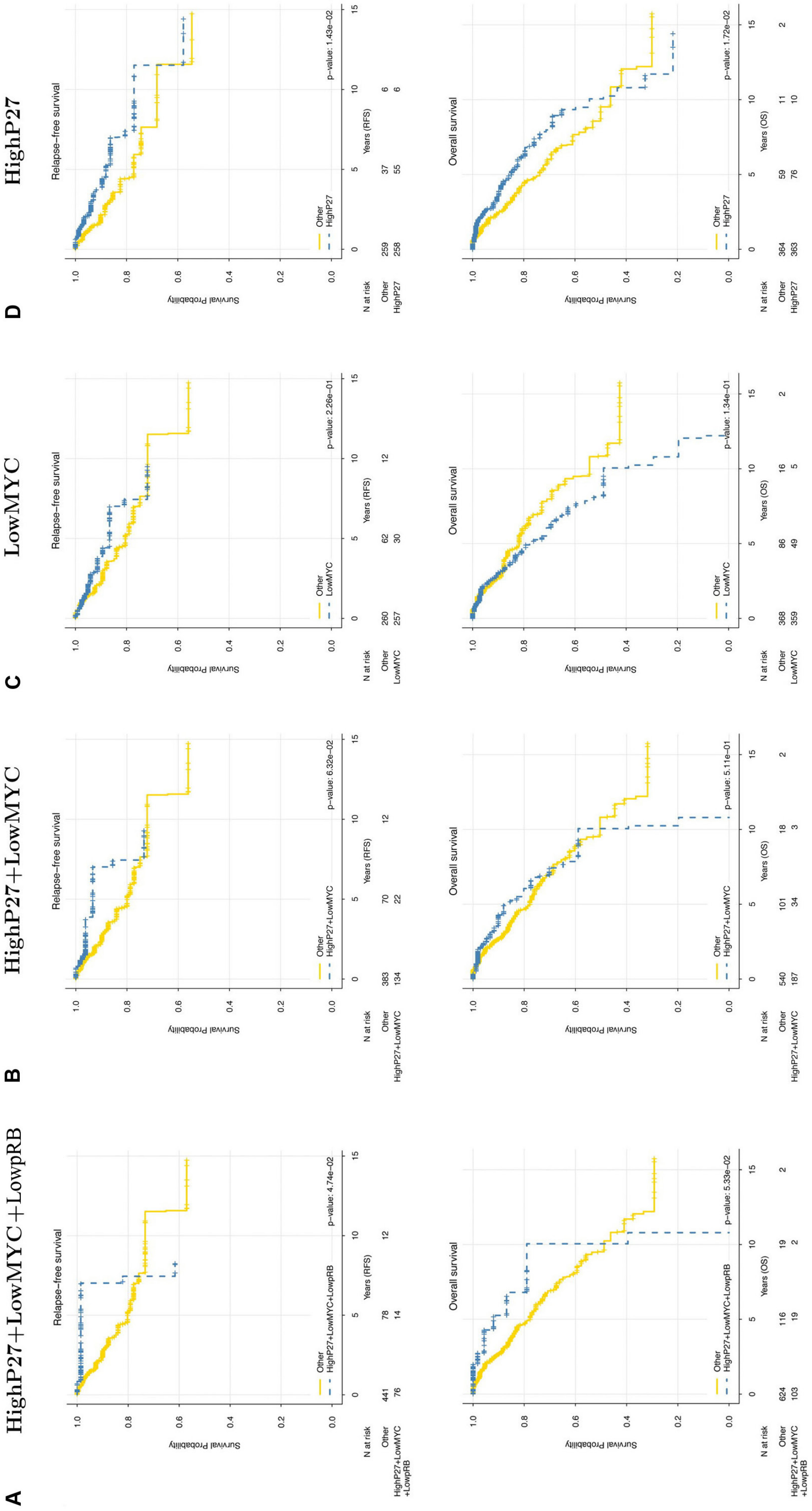

Supplementary Figure S5: Kaplan-Meier presentation of relapse-free and overall survival in breast invasive carcinoma populations with a combination of (A) high p27+low Myc+low pRb, (B) high p27+low Myc, (C) low Myc and (D) high p27.

Supplementary Table S1

HighP27+LowMYC+LowP27T157

Clinical Parameters

Pathologic State

Group	Other (%)	HighP27+LowMYC+LowP27T157 (%)
Stage I	13.57	25.44
Stage II	58.48	57.89
Stage III	25.69	15.79
Stage IV	2.26	0.88
Total %	100.00	100.00
Total Number	619	114

Molecular subtype

Group	Other (%)	HighP27+LowMYC+LowP27T157 (%)
Basal	20.21	2.73
Her2	10.10	1.82
LumA	42.81	72.73
LumB	24.14	20.91
Normal	2.74	1.82
Total %	100.00	100.00
Total Number	584	110

ER Status

Group	Other (%)	HighP27+LowMYC+LowP27T157 (%)
Negative	28.38	1.83
Positive	71.62	98.17
Total %	100.00	100.00
Total Number	592	109

HER2 Status (nature 2012)

Group	Other (%)	HighP27+LowMYC+LowP27T157 (%)
Negative	83.14	92.52
Positive	16.86	7.48
Total %	100.00	100.00
Total Number	516	107

Supplementary Table S2

HighP27+LowMYC+LowpRB

Clinical Parameters

Pathologic State

Group	Other (%)	HighP27+LowMYC+LowpRB (%)
Stage I	14.04	23.58
Stage II	59.17	53.77
Stage III	24.56	21.70
Stage IV	2.23	0.94
Total %	100.00	100.00
Total Number	627	106

Molecular subtype

Group	Other (%)	HighP27+LowMYC+LowpRB (%)
Basal	19.46	5.83
Her2	9.98	1.94
LumA	43.99	67.96
LumB	24.03	21.36
Normal	2.54	2.91
Total %	100.00	100.00
Total Number	591	103

ER Status

Group	Other (%)	HighP27+LowMYC+LowpRB (%)
Negative	27.41	5.05
Positive	72.59	94.95
Total %	100.00	100.00
Total Number	602	99

HER2 Status (nature 2012)

Group	Other (%)	HighP27+LowMYC+LowpRB (%)
Negative	84.03	88.66
Positive	15.97	11.34
Total %	100.00	100.00
Total Number	526	97

Supplementary Table S3

HighP27+LowMYC

Clinical Parameters

Pathologic State

Group	Other (%)	HighP27+LowMYC (%)
Stage I	13.15	21.76
Stage II	58.52	58.03
Stage III	25.93	19.17
Stage IV	2.41	1.04
Total %	100.00	100.00
Total Number	540	193

Molecular subtype

Group	Other (%)	HighP27+LowMYC (%)
Basal	20.87	8.06
Her2	10.83	3.23
LumA	41.73	63.44
LumB	23.82	23.12
Normal	2.76	2.15
Total %	100.00	100.00
Total Number	508	186

ER Status

Group	Other (%)	HighP27+LowMYC (%)
Negative	30.06	7.69
Positive	69.94	92.31
Total %	100.00	100.00
Total Number	519	182

HER2 Status (nature 2012)

Group	Other (%)	HighP27+LowMYC (%)
Negative	83.07	89.08
Positive	16.93	10.92
Total %	100.00	100.00
Total Number	449	174

Supplementary Table S4

LowMYC

Clinical Parameters

Pathologic State

Group	Other (%)	LowMYC (%)
Stage I	13.35	17.49
Stage II	58.04	58.74
Stage III	26.70	21.58
Stage IV	1.91	2.19
Total %	100.00	100.00
Total Number	367	366

Molecular subtype

Group	Other (%)	LowMYC (%)
Basal	23.24	11.86
Her2	7.35	10.17
LumA	46.18	48.87
LumB	20.29	26.84
Normal	2.94	2.26
Total %	100.00	100.00
Total Number	340	354

ER Status

Group	Other (%)	LowMYC (%)
Negative	30.20	18.29
Positive	69.80	81.71
Total %	100.00	100.00
Total Number	351	350

HER2 Status (nature 2012)

Group	Other (%)	LowMYC (%)
Negative	87.15	82.69
Positive	12.85	17.31
Total %	100.00	100.00
Total Number	288	335

Supplementary Table S5

HighP27

Clinical Parameters

Pathologic State

Group	Other (%)	HighP27 (%)
Stage I	12.81	18.03
Stage II	58.86	57.92
Stage III	25.61	22.68
Stage IV	2.72	1.37
Total %	100.00	100.00
Total Number	367	366

Molecular subtype

Group	Other (%)	HighP27 (%)
Basal	22.19	12.68
Her2	13.54	4.03
LumA	34.29	60.81
LumB	27.95	19.31
Normal	2.02	3.17
Total %	100.00	100.00
Total Number	347	347

ER Status

Group	Other (%)	HighP27 (%)
Negative	33.05	15.43
Positive	66.95	84.57
Total %	100.00	100.00
Total Number	351	350

HER2 Status (nature 2012)

Group	Other (%)	HighP27 (%)
Negative	78.91	90.65
Positive	21.09	9.35
Total %	100.00	100.00
Total Number	313	310
